# Implementation context, mechanisms and outcomes of a transitional care intervention to prevent delirium: a mixed-methods process evaluation from the TRADE study

**DOI:** 10.1186/s12877-025-06331-8

**Published:** 2025-09-25

**Authors:** Natascha-Elisabeth Denninger, Simone Brefka, Gabriele Meyer, Marlene Benkert, Dhayana Dallmeier, Michael Denkinger, Martin Müller

**Affiliations:** 1 Department of Primary Care and Health Services Research, Heidelberg University/University Hospital Heidelberg, Medical Faculty Heidelberg, Nursing Science and Interprofessional Care, Im Neuenheimer Feld 130.3, Heidelberg, 69120 Germany; 2 International Graduate Academy, Martin Luther University Halle-Wittenberg, Institute of Health, Midwifery and Nursing Science, Medical Faculty, Magdeburger Straße 8, Halle (Saale), 06112 Germany; 3 Centre for Research, Development and Technology Transfer, Rosenheim Technical University of Applied Sciences, Hochschulstraße 1, Rosenheim, 83024 Germany; 4 Agaplesion Bethesda Hospital Ulm, Research Unit on Ageing, Zollernring 26, Ulm, 89073 Germany; 5 Geriatric Centre Ulm at Agaplesion Bethesda Hospital Ulm, Zollernring 26, Ulm, 89073 Germany; 6 Institute for Geriatric Research, University Hospital Ulm at Agaplesion Bethesda Hospital Ulm, Zollernring 26, Ulm, 89073 Germany; 7 Department of Epidemiology, Boston University School of Public Health, 715 Albany Street, Boston, MA 02118 USA

**Keywords:** Delirium prevention, Older patients, Caregivers/Family, Transitional care, Patient discharge, Process evaluation, Mixed-methods study

## Abstract

**Background:**

While predisposing factors for delirium, like old age or surgery, are well documented, less attention has been paid to environmental factors, including hospital transfer processes and caregiver involvement in transitional care of older patients. To address this gap, we developed a pathway to optimize hospital transfer processes and actively involve caregivers in preventing delirium. This complex intervention was tested in a pilot study using a stepped-wedge design across four hospitals, accompanied by a process evaluation to explore the implementation context, mechanisms and outcomes of this intervention.

**Methods:**

A parallel convergent mixed-methods process evaluation was used. Qualitative data and quantitative data were analyzed separately and integrated using a weaving approach. Analyses were guided by Normalization Process Theory, supplemented by implementation impact ratings based on the Consolidated Framework for Implementation Research rating tool.

**Results:**

Data included 72 interviews, 2 focus groups, 82 document analyses, 14 status analyses, 424 TRADE questionnaires, 58 Normalization MeAsure Development questionnaires, and website traffic metrics. COVID-19-related constraints resulted in partial implementation of the intervention, with challenges such as limited training opportunities and restricted caregiver involvement. Healthcare professionals reported greater delirium awareness, and educational materials received positive feedback.

**Conclusion:**

The study underscores the critical role of discharge information, post-discharge support, and education for caregiver and healthcare professionals in preventing delirium. It also provides evidence of how the COVID-19 pandemic impacted standard care and the implementation of clinical interventions, emphasizing the need for adaptable processes and institutional support. Furthermore, it offers theoretical and methodological insights into conducting mixed-methods process evaluations in complex intervention research.

**Trial registration:**

German Clinical Trials Register (ID: DRKS00017828, retrospectively registered on 17.09.2019, https://drks.de/search/en/trial/DRKS00017828).

**Supplementary Information:**

The online version contains supplementary material available at 10.1186/s12877-025-06331-8.

## Background

Delirium is a neurocognitive disorder characterized by an acute disturbance in attention, awareness, and cognition. It often manifests with disorientation, language impairments, and memory deficits, and is known to fluctuate over the course of the day. Three subtypes of delirium are typically distinguished: hyperactive, hypoactive, and mixed [1]. Hypoactive delirium is especially underdiagnosed due to its subtle presentation [2]. Diagnostic challenges are further compounded by symptom fluctuation [3], the lack of baseline cognitive assessments, and difficulty conducting cognitive testing in hospital settings [4].

Both prevalence and incidence of delirium vary widely depending on the setting. International estimates indicate rates of 17–35% upon hospital admission in surgical and medical wards, and up to 50% in intensive care units [5]. In long-term care facilities, prevalence rates of up to 37% have been reported [6]. Data from German-speaking countries offer further insight into the regional epidemiology: a recent secondary data analysis conducted in Germany, Austria, and Switzerland identified a point prevalence of 7.1% in the morning and 7.2% in the evening across general hospital wards, emergency departments, rehabilitation centers, and nursing homes [7]. Incidence rates range from 11–51% in surgical and medical wards and increase to 19–82% in intensive care units [5]. Delirium is associated with negative outcomes, such as prolonged hospital stays, functional decline, nursing home admissions, increased caregiver burden [8, 9], and higher mortality [9, 10].

Well-documented predisposing risk factors for delirium include advanced age, pre-existing cognitive impairment, sensory deficits, and malnutrition. Common precipitating factors are surgical procedures, medication and acute illness [11].

To prevent delirium, avoiding and reducing risk factors, as well as implementing multicomponent interventions, have proven particularly effective [12, 13]. Structured programs such as the Hospital Elder Life Program (HELP) exemplify effective non-pharmacological strategies for delirium prevention by targeting modifiable risk factors through multicomponent interventions. These components include cognitive stimulation, early mobilization, and (re-)orientation [14, 15]. HELP significantly reduces the incidence of delirium as demonstrated by a meta-analysis [16]. Building on this approach, Family-HELP extends the intervention by actively involving patients’ close relatives to enhance orientation and communication. Family-HELP includes discussing past and future events and providing regular reminders to use visual and hearing aids to support sensory input [17]. The involvement of caregivers is further supported by other studies, which highlight their potential to detect early signs of cognitive changes and assist with patient orientation in unfamiliar hospital environments [18, 19].

Environmental stressors such as frequent bed or room changes, particularly during hospital transfers, are also recognised as significant triggers for delirium. Goldberg et al. (2015) [20] and McCusker et al. (2001) [21] showed that an increased number of room changes was associated with higher delirium incidence. More than 90% of hospital patients experience transfers during their stay [22], yet delirium prevention strategies often neglect the vulnerable periods before, during, and after such transitions. Although studies include follow-up or continue interventions after transition [23, 24], none have targeted these transitional phases through structured prevention approaches.

To address this gap, within the TRAnsport and DElirium in older adults (TRADE) study, a complex intervention for preventing delirium in older hospitalized patients has been developed and tested to optimize discharge and transfer procedures and foster the active engagement of caregivers (family members, close friends of patients) before, during, and after these transitions [25, 26]. This intervention was tested in a pilot study using a stepped-wedge design in four German hospitals. The predefined process evaluation accompanying the pilot study is detailed in this article, while the analysis of the principal outcome will be published separately.

Process evaluations are critical for understanding complex interventions [27]. To capture the dynamic and multifaceted nature of such interventions, process evaluations must address diverse research questions, necessitating both qualitative and quantitative research methods [28, 29]. This mixed-methods approach enables researchers to determine not only *whether* the developed intervention works, but also to explore *how* and *why* it succeeds or fails [30]. Furthermore, this form of evaluation enables a comprehensive understanding of the factors influencing the intervention and its effectiveness, providing a foundation for further development of the intervention and its implementation strategies [31].

This article presents the mixed-methods process evaluation for the TRADE intervention study, examining the program theory and theoretical assumptions underpinning the TRADE intervention [25]*,* alongside the implementation context, facilitators, and barriers. This approach follows the Medical Research Council (MRC) framework for developing and evaluating complex interventions [29].

## Methods

### Aims and research questions

The process evaluation aimed to explore the feasibility of the designed complex intervention and its implementation strategies, to assess its implementation, and to identify relevant contextual factors. The predefined research questions were as follows:

#### Implementation context

- ▪ What was the context in which the intervention was implemented?
- ▪ What contextual factors facilitated or hindered the intervention’s implementation?

#### Implementation mechanisms and outcomes

- ▪ Was the intervention implemented as planned?
- ▪ How were the intervention and implementation strategies executed (dose delivered/received, fidelity, implementation)?
- ▪ What factors facilitated or hindered the implementation of the intervention?
- ▪ How did healthcare professionals experience the implementation process? How acceptable, appropriate, and feasible did they rate the TRADE intervention?
- ▪ Which change mechanisms were observed?

### The TRADE intervention

The intervention comprises an 8-point discharge pathway designed to prevent delirium by offering guidance for caregivers assisting patients before, during, and after hospital discharges or transfers. This pathway includes: (1) encouraging caregiver presence before, during and after discharge, (2) fostering familiarity, (3) conveying information, (4) aiding orientation, (5) adapting communication, (6) structuring daily routines, (7) promoting physical activity, and (8) ensuring proper nutrition. Healthcare professionals engaged caregivers of patients aged 70 years or older at least 48 h before discharge through conversation, flyers, and online videos. Information about discharge and post-discharge care was also provided to patients and follow-up care providers. The implementation was supported by gatekeepers, champions, and contact persons in participating wards, who facilitated and assessed the intervention and provided ongoing professional assistance for healthcare professionals and the study team. Champions received monthly mentoring from the research team. Resources such as handbooks, posters, and videos with information on delirium, program details, conversation guides, and checklists were distributed amongst healthcare professionals. Leaders of the participating hospitals were also informed about the intervention to encourage institutional support [25]. Table 1 provides a detailed overview of the intervention components.

**Table 1:** Overview of the TRADE intervention

Component	Content	Target group
Preparing discharge/transfer	- Caregivers: Information on 8-point program (via conversations, flyers, and videos) - Patients: Details of discharge/transfer process and post-discharge/transfer planning (via conversations) - Healthcare professionals: Further actions including notifying follow-up care providers about discharge date and patient information, providing discharge letters, and scheduling discharge between the hours of 7 am and 6 pm	- Patients aged 70 + - Caregivers - Follow-up care providers
Gatekeepers	- Supporting the implementation within hospitals by acting as influencers, gaining in-depth knowledge of processes, and identifying champions - Status analysis, creating posters with “Dos and don´ts”	- Healthcare professionals (preference leaders)
Champions and contact persons	- Champions: Serving as direct contacts for the study team, as liaisons between the ward and the study team, and as multipliers - Contact persons: Due to the pandemic, two clusters could not provide champions and instead nominated contact persons, who partially relayed information to the wards and provided limited support for implementation - Both received feedback contact, with mentoring exclusive to champions	- Healthcare professionals
Status analysis	- Identifying local structures, local experts on delirium, and discharge processes in the hospitals, and surveying change processes through the intervention components and implementation strategies	- Each ward/department of the participating hospitals - Gatekeepers/champions
Training	- Champions/gatekeepers: Qualifying the champions and gatekeepers to pass on their knowledge to their teams and implement the TRADE intervention (study team) - Healthcare professionals: Qualifying the ward teams to conduct and implement the TRADE intervention (champions)	- Champions and gatekeepers - Healthcare professionals
Information materials	- Healthcare professionals receive: posters, handbook, conversation guide - Caregivers receive: flyers and videos	- Healthcare professionals
Information to leaders	- Providing leaders with implementation support	- Leaders (interprofessional)

### Design

We conducted a mixed-methods process evaluation using a convergent parallel design [32, 33]. The study protocol for the pilot study and the process evaluation has been published previously [26]. Our evaluation was based on a pre-developed program theory [25]*,* which provided theoretical underpinnings to facilitate planning and presentation of inputs/resources, activities, outputs, outcomes and impact. Normalization Process Theory (NPT) served as a theoretical and conceptual foundation to understand and examine the process of implementing new practices [34, 35]. NPT focuses on three key areas: *Implementation Context*, *Mechanisms*, and *Outcomes* [34].

We analyzed the experiences of healthcare professionals, patients, and caregivers described in interviews and focus groups in conjunction with quantitative findings, enhancing our comprehension of the practical aspects of the study components. Qualitative methods comprised: (a) semi-structured interviews (patients, caregivers, healthcare professionals, coordinating study nurses and study physicians); (b) focus groups (healthcare professionals); (c) document analyses; and (d) status analyses of each specialized cluster department (including discharge processes, trainings). Quantitative methods included: (e) TRADE questionnaire (patients, caregivers); (f) the Normalization MeAsure Development (NoMAD) questionnaire [35–37] from NPT (healthcare professionals); and (g) monitoring of the intervention website page views.

Reporting followed the guidelines outlined in the Good Reporting of a Mixed Methods Study (GRAMMS) [38] (see Supplementary file 1).

### Setting and sampling

The study was conducted across three university hospitals and one geriatric hospital in South-Western Germany, with a total of 18 wards (~ 400 beds) organized into four clusters pre-defined at the time of the funding application. Participating medical disciplines included: internal medicine (five cardiology wards, one cardiology/gastroenterology ward, one gastroenterology/endocrinology ward, one oncology ward, two wards with mixed specializations), geriatrics (three geriatric wards, one geriatric/gastroenterology ward) and traumatology (four wards).

To obtain comprehensive data for the research questions, a combination of convenience and purposive sampling approaches was employed.

### Data collection and analysis

The trial was conducted between April 2021 and March 2022 (52 weeks). All four clusters began the trial without the intervention (t1; control phase) and then started the intervention in a staggered manner, with all clusters completing the intervention simultaneously. The intervention phase (t2) started as follows: Cluster 1 in week 13, Cluster 2 in week 19, Cluster 3 in week 25, and Cluster 4 in week 31.

The process evaluation was carried out at multiple time points (t0–t3) between February 2021 and May 2022. Data collection began at t0, before the trial began, and continued at t1 during the control phase, at t2 during the intervention phase, and at t3 immediately after the intervention’s conclusion. Due to the stepped-wedge design, it was crucial to establish consistent data collection intervals relative to the intervention’s start in each cluster to ensure comparability and avoid bias. To assess changes occurring throughout the intervention phases, detailed data were collected for each phase [39]. Period t2 thus consisted of the following time points for process evaluation: t2a (weeks 4 and 5), t2b (weeks 11 and 12), t2c (weeks 12 to 20), t2d (weeks 29 and 30), and t2e (two weeks before the intervention phase ended). Two weeks were allotted for the NoMAD questionnaire per data collection period (t2a, t2b, t2d, and t2e), while eight weeks were allocated for interviews and focus groups with healthcare professionals (t2c). Table 2 summarizes the methods, datasets, and time points used in the mixed-methods process evaluation.

**Table 2:**
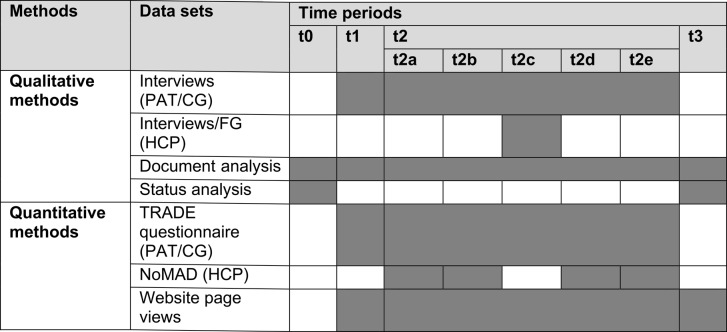
Summary of data collection methods, data sets, and time points

*PAT* Patients, *CG *Caregivers, *FG *Focus groups, *HCP *Healthcare professionals

t0: Pre-study; t1: Control phase; t2: Intervention phase, with t2a: Intervention weeks 4 and 5; t2b:Intervention weeks 11 and 12; t2c: Intervention weeks 12 to 20; t2d: Intervention weeks 29 and30; t2e: 2 weeks before the end of the study (Intervention weeks: Cluster 1 weeks 38 and 39,Cluster 2 weeks 32 and 33, Cluster 3 weeks 26 and 27, Cluster 4 weeks 20 and 21); t3: Post-study

*nurses, physiotherapists, speech/occupational therapists, physicians, administrative staff, persons responsible for patient/discharge management, and social workers); NoMAD: Normalization Measure Development

#### Qualitative data collection and analyses

(a) Interviews and (b) focus groups: For interviews and focus groups with healthcare professionals, we aimed for 100% inclusion of gatekeepers, champions, contact persons, and coordinating study nurses and study physicians to ensure representation of all relevant professional groups and seniority levels. Additional healthcare professionals were included until data saturation was reached, as determined by consensus between the two coders of the data. All patients and caregivers who completed the TRADE questionnaire (e) were invited to participate in follow-up interviews (a). The qualitative element of this study was theoretically grounded in the NPT [34] and the predeveloped program theory of the TRADE intervention [25]. Semi-structured, guided interviews and focus groups were designed based on the core elements of NPT (Coherence, Cognitive participation, Collective Action, and Reflexive Monitoring) [34] and tailored to the contextual dimensions of the program theory (see Supplement 2).

Interviews were conducted via telephone. Focus groups with healthcare professionals were held online using a data protection-compliant videoconferencing system. All sessions were systematically moderated, and supplemented by note-taking. The interview guides were pretested with healthcare professionals, and minor adjustments were made to improve question phrasing. All interviews and focus groups were conducted in German language, audio-recorded, and transcribed verbatim using the transcription software f4transkript (version 2021) [computer software, dr. dresing & pehl GmbH].

(c) Document analysis: Meetings with the study team and healthcare professionals, including (peer-mentoring) sessions with key individuals such as gatekeepers, champions, and contact persons, were documented in detail. Special attention was given to the topics discussed, study planning adjustments, pandemic-related changes, and resulting decisions. The documentation included minutes from meetings with cluster staff, study partners, and management teams, as well as records of discussions with champions and contact persons (one set of minutes per cluster for the entire study period).

(d) Status analysis: Standardized forms for status analysis, based on the German Expert Standard for Discharge Management [40], were completed for each department. These forms included additional questions about departmental and ward characteristics (see Supplement 3). These baseline and follow-up analyses were analyzed using Microsoft Excel 2021 (Version 16.0) [computer software, Microsoft Corporation], allowing for comparison over time.

Qualitative data were analyzed using content analysis [41], with MAXQDA (version 2020) [computer software, VERBI GmbH] employed for data handling. Document analysis was conducted in parallel with the interviews and focus groups. Status analyses were comparatively examined and integrated using the NPT coding manual [42]. Deductive codes were derived from Parts A and B of the manual, with additional inductive codes, for example for the evaluation of informational materials (flyers, videos, etc.). An initial coding manual was developed by a researcher experienced in qualitative methods (ND), reviewed and tested by a second researcher (MB), and subsequently discussed. Sample size was determined by data saturation, reached by consensus between two researchers when no new information emerged. German quotations were translated into English for reporting.

#### Quantitative data collection and analyses

(e) TRADE questionnaire: Based on sample size calculations, the TRADE cRCT aimed to recruit a total of 500 patients and caregivers across all clusters (approximately 125 participants per cluster within a 12-month period). Eligible patients were aged 70 years or older with a planned discharge within three days. Exclusion criteria were patients receiving palliative care, with a prognosis of less than three months survival, and the presence of delirium at the time of inclusion, defined by the 3-Minute Diagnostic Interview for Confusion Assessment Method-defined Delirium (3D-CAM) [43], and the Nursing Delirium Screening Scale (Nu-DESC) [44, 45]. Due to the fluctuating nature of delirium, some cases may not have been identifiable at the time of recruitment but were retrospectively excluded based on later clinical assessments or documentation. Cognitive impairment was considered either known, based on existing diagnoses, or suspected through clinical interaction or hospital documentation. If patients lacked the capacity to provide informed consent due to cognitive impairment (e.g., dementia) and no legally authorized representative (LAR) was available, they were excluded from participation. If available, consent was obtained from the LAR [26].Quantitative data were collected by trained study nurses using a web-based electronic data capture (EDC) system, secuTrial® (version 6.1.1.8) [computer software, interActive Systems GmbH], with data primarily gathered in person, or by telephone where necessary. Closed questions were used for process evaluation (see Supplement 4). Scientific data managers continuously reviewed the eCRF database for inconsistencies or discrepancies, which they directly queried with study nurses from each cluster. Statistical analyses were performed using IBM SPSS Statistics (Version 28.0) [computer software, IBM Corporation]. Data analysis included absolute frequencies for categorical variables and exploratory group comparisons between intervention and control groups via chi-square (χ^2^) test; Fisher's exact test was applied for smaller samples [46].

(f) NoMAD: All healthcare professionals on the participating wards were invited to complete the NoMAD questionnaire [35–37], with invitations distributed via email. The NoMAD questionnaire together with additional questions (see Supplement 5) were administered using QuestorPro (Version 4.1) [computer software, Blubbsoft GmbH]. Healthcare professionals received information and login details via email. Data were analyzed in Microsoft Excel 2021 (Version 16.0) [computer software, Microsoft Corporation], with responses summarized and evaluated according to absolute frequencies [47], following the recommended approach [37].

Website views: Access to the study website was tracked using a web analytics tool, which recorded access from each browser from the start of the study to completion. Data analysis in Microsoft Excel 2021 (Version 16.0) included comparison of website traffic with key events, such as the intervention’s initiation in each cluster.

#### Synthesis

Qualitative and quantitative data were collected concurrently and initially analyzed separately. To explore the implementation context, qualitative data underwent method triangulation [48]. In a final step, categories of the implementation context were assessed using the Consolidated Framework for Implementation Research (CFIR) rating tool [49] to evaluate their impact on the intervention's implementation. The influence was indicated as follows: “ + ” (positive/facilitating), “–” (negative/barrier), “X” (mixed), and “0” (neutral). The strength of each influence was rated as “1” (weak) or “2” (strong).

To gain a comprehensive understanding of the implementation mechanisms and outcomes, qualitative and quantitative results were integrated using a weaving approach within a convergent, parallel mixed-methods design [32, 33]. Joint displays were used to thematically organize and present qualitative findings (narrative form) alongside quantitative data (descriptive form and figures). Each theme was evaluated using the CFIR tool, enabling categorization by theme, direct comparison, and meta-inference. The integrated results are summarized narratively in the results section.

Lastly, the process evaluation results were applied to review the logic model for program implementation. Where necessary, the model was adjusted based on these findings. The results were also used to assess the implementation status of individual components and color-coded as follows: green (primarily implemented), orange (partially implemented), red (primarily not implemented), and blue (not applicable).

## Results

### Characteristics of the sample

#### Qualitative study parts

(a) Interviews and (b) focus groups: A total of 72 individual interviews and two focus groups were conducted. All consenting patients and caregivers participated, with interviews including 16 participants at t1 (10 patients and 6 caregivers) and 17 at t2 (9 patients and 8 caregivers). These 33 interviews lasted 964 min in total (range: 9–66 min). A total of 39 interviews were conducted with healthcare professionals, including five nurses, two physicians, two secretaries, one social services staff member, nine champions (4 nurses, 3 physicians, 2 social services staff), five contact persons (2 nurses, 1 patient management staff, 1 physiotherapist, 1 occupational therapist/speech therapist), four gatekeepers (all physicians), four nursing/department managers, four study assistants, and three study physicians. All targeted groups (gatekeepers, champions, contact persons, coordinating study assistants) were successfully recruited, except for study physicians, with just three of the targeted four. These interviews had a total duration of 1,522 min (range: 20–66 min). Two focus groups, each with five participants (3 nursing contact persons, 2 medical directors), lasted 81 min in total (range: 40–41 min). Data saturation was achieved.

(c) Document analysis: A total of 82 documents were analyzed.

(d) Status analysis: Fourteen status analyses were conducted, seven at t0 and seven at t3.

#### Quantitative study parts 

(e) TRADE questionnaire: We recruited 396 individuals (control: 181, intervention: 215). A total of 80 participants terminated the study early, and 104 were excluded due to suspected delirium or missing data. Since some prevalent cases of delirium at baseline were identified retrospectively through reviewing patient records (see Quantitative data collection and analyses), the final sample comprised 212 participants (control: 94, intervention: 118) for the process evaluation study (see flowchart in Supplement 6).

(f) NoMAD: To address low response rates, data collection intervals for the NoMAD questionnaire were adjusted, extending the t2a and t2e phases from two weeks to four and omitting phases t2b and t2d. In total, 58 questionnaires were completed (t2a: *n* = 29; t2e: *n* = 29), yielding an approximate response rate of 5.8% for each period, with around 500 healthcare professionals invited each time (t2a; t2e). Note that this figure is an estimate due to staff turnover rates. Incomplete responses (*n* = 129, 12,9%) were excluded. At t2a, respondents included 19 nurses, one nursing assistant, one Advanced Practice Nurse (APN), four physicians, one secretary, one physiotherapist, one speech therapist, and one social services staff member. At t2e, they included 16 nurses, one APN, six physicians, two secretaries, one physiotherapist, one speech therapist, and two social services staff members.

A detailed overview of the sample characteristics at each timepoint and participant details for the qualitative and quantitative study components is provided in Supplement 7.

### Evaluation of Implementation Context

The *Implementation Context* encompasses the organizational conditions and factors influencing how the intervention was adopted and sustained. Subcategories include *Reframing Organizational Logics*, *Strategic Intentions*, *Negotiating Capacity*, and *Adaptive Executions*.

The intervention’s implementation encountered considerable challenges due to structural limitations and pandemic-related disruptions. Negative perceptions of the term "study", competing priorities in certain wards, and limited email communication hindered participation. Pre-existing issues such as staff shortages and high turnover were exacerbated by COVID-19, which further strained resources and disrupted hospital routines. Visitor restrictions affected caregiver involvement (see Supplement 8) and access to informational materials. To mitigate these challenges, adaptations to the intervention were necessary: rather than appointing champions in each cluster, some clusters designated contact persons. However, their ability to support implementation was limited. Training sessions were shortened and delivered online, employing various formats to facilitate knowledge dissemination across wards, such as in-person and online sessions, on-demand recordings, multiple sessions, self-study materials, and additional information via emails. Despite these adaptations, the implementation context, deeply impacted by the pandemic, exerted a strongly negative influence on the intervention (–/2). Table 3 summarizes these influencing factors and their ratings, with example quotes provided in Supplement 9.

**Table 3:**
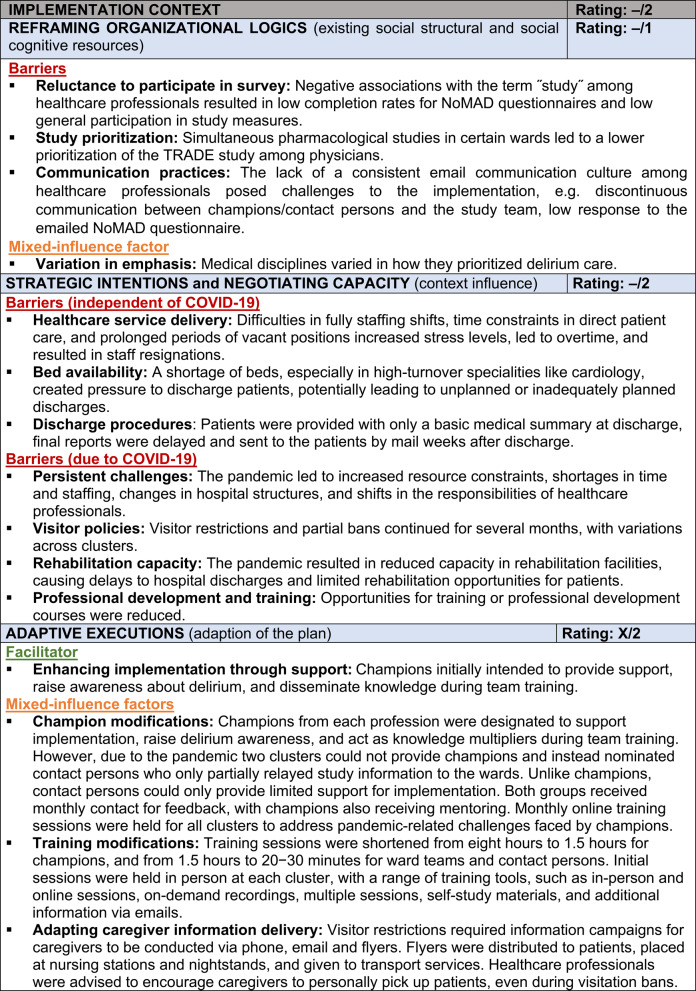
Overview of influencing factors and ratings of the Implementation Context

Legend: Influence rating: + = positive influence (facilitator), – = negative influence (barrier), X = mixed influences, 0 = neutral; Strength of influence: 1 = weak influence, 2 = strong influence on implementation

### Evaluation of Implementation Mechanisms

*Implementation Mechanisms* describe the processes and interactions involved in integrating the intervention into existing structures, focusing on *Coherence*, *Cognitive Participation*, *Collective Action*, and *Reflexive Monitoring*.

#### Coherence

*Coherence* assesses how participants understand and perceive the relevance of the TRADE intervention within their roles, covering *Differentiation*, *Internalization*, *Communal Specification*, and *Individual Specification*. Reception varied across medical disciplines: while geriatrics demonstrated high acceptance, disciplines like cardiology and oncology exhibited lower receptivity, leading to mixed effects on intervention differentiation and internalization. Communal specification was largely positive, as diverse training formats facilitated knowledge dissemination and enhanced awareness of delirium prevention. However, role ambiguities, particularly for champions and study nurses, along with limited interprofessional collaboration created implementation challenges. Thus, *Coherence* had a mixed impact (X/2): while knowledge dissemination and shared understanding were effective, role clarity and acceptance in some disciplines posed challenges. Table 4 presents the joint display for *Coherence*, with example quotes from the qualitative data provided in Supplement 9.

**Table 4:**
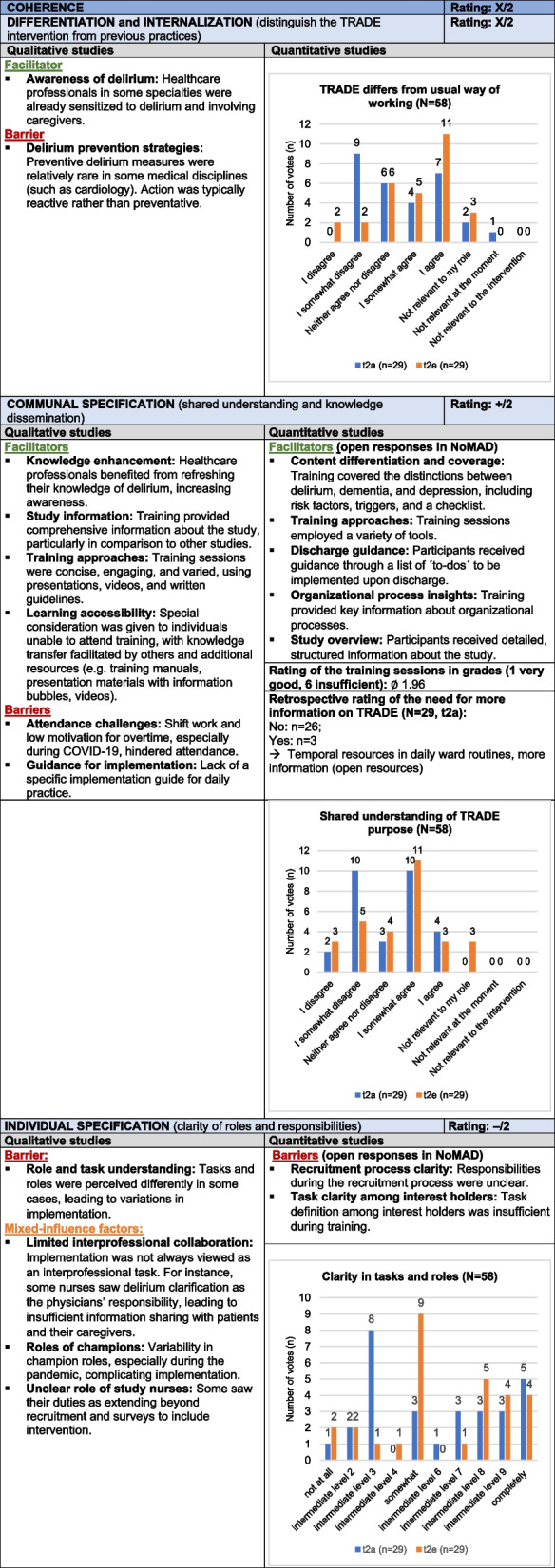
Joint display of *Coherence*

Influence rating: + = positive influence (facilitator), – = negative influence (barrier), X = mixed influences, 0 = neutral; Strength of influence: 1 = weak influence, 2 = strong influence on implementation

#### Cognitive Participation

*Cognitive Participation* examines the collaborative effort in implementing the intervention, with subcategories *Initiation*, *Enrollment*, *Legitimation*, and *Activation*. Early engagement from department leaders and the presence of champions facilitated the initial stages. However, barriers arose in two clusters where leaders limited the champions’ efforts, and some wards perceived the program as additional workload. Competing studies and inconsistent communication practices, particularly among physicians, led to issues such as unanswered emails and difficulties in ensuring consistent collaboration. Despite these obstacles, many participants supported the intervention dissemination, reflecting mixed engagement and significant challenges (X/2). Table 5 presents the joint display of *Cognitive Participation*, with example quotes from the qualitative data provided in Supplement 9.

**Table 5:**
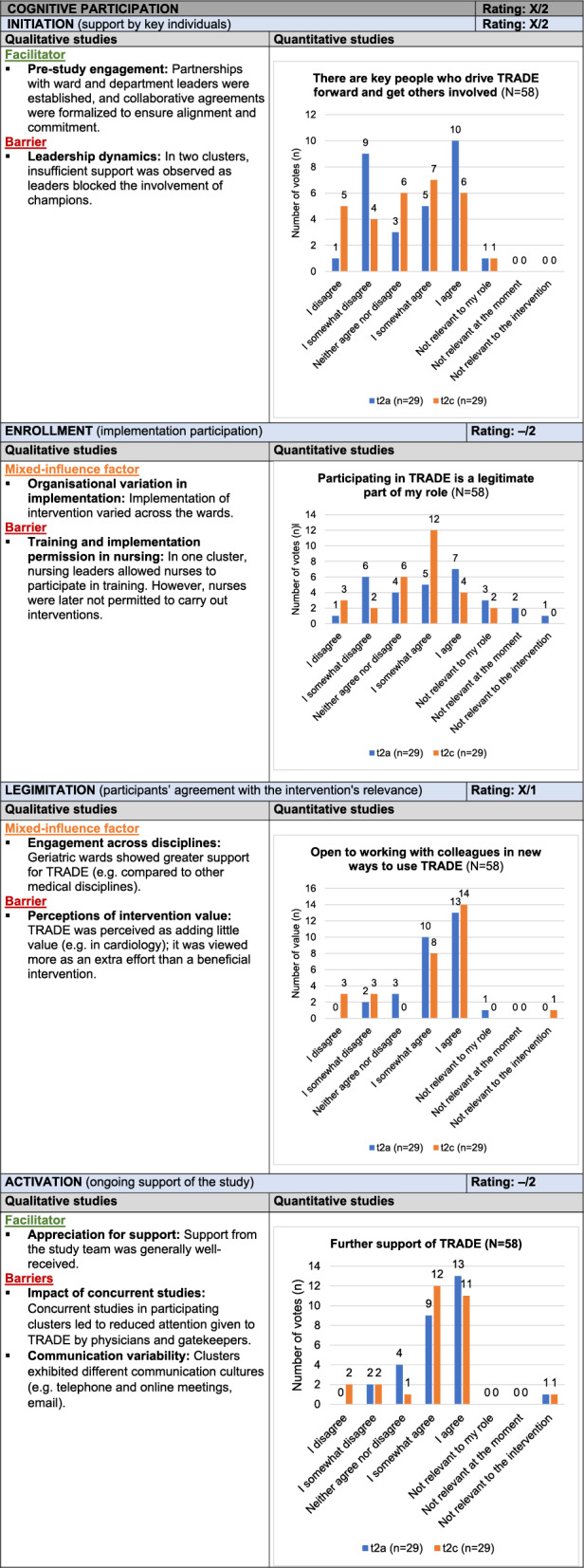
Joint display of C*ognitive Participation*

Influence rating: + = positive influence (facilitator), – = negative influence (barrier), X = mixed influences, 0 = neutral; Strength of influence: 1 = weak influence, 2 = strong influence on implementation

#### Collective Action

*Collective Action* focuses on the coordinated effort within the organization to realize the intervention, including *Interactional Workability*, *Relational Integration*, *Skill Set Workability*, and *Contextual Integration*. COVID-19 pandemic related restrictions significantly impacted the implementation, limiting caregiver involvement and reducing access to information. The distribution of flyers was limited, with most reaching patients rather than caregivers, while online resources for caregivers remained underutilized, diminishing their reach and impact. Teamwork varied: some clusters incorporated the intervention into daily discussions, while others lacked interprofessional collaboration. Training materials supported knowledge retention effectively. Management expressed interest in progress during regular meetings, yet additional resources or time for champions were not provided, partially hindering implementation. Inconsistent organizational support and the absence of sanctions for non-implementation limited engagement. Collectively, pandemic-related restrictions, team coordination issues, and limited organizational support created challenges, though training materials contributed positively, resulting in a mixed rating (X/2). Table 6 presents the joint display of *Collective Action*, with example quotes from the qualitative data provided in Supplement 9.

**Table 6:**
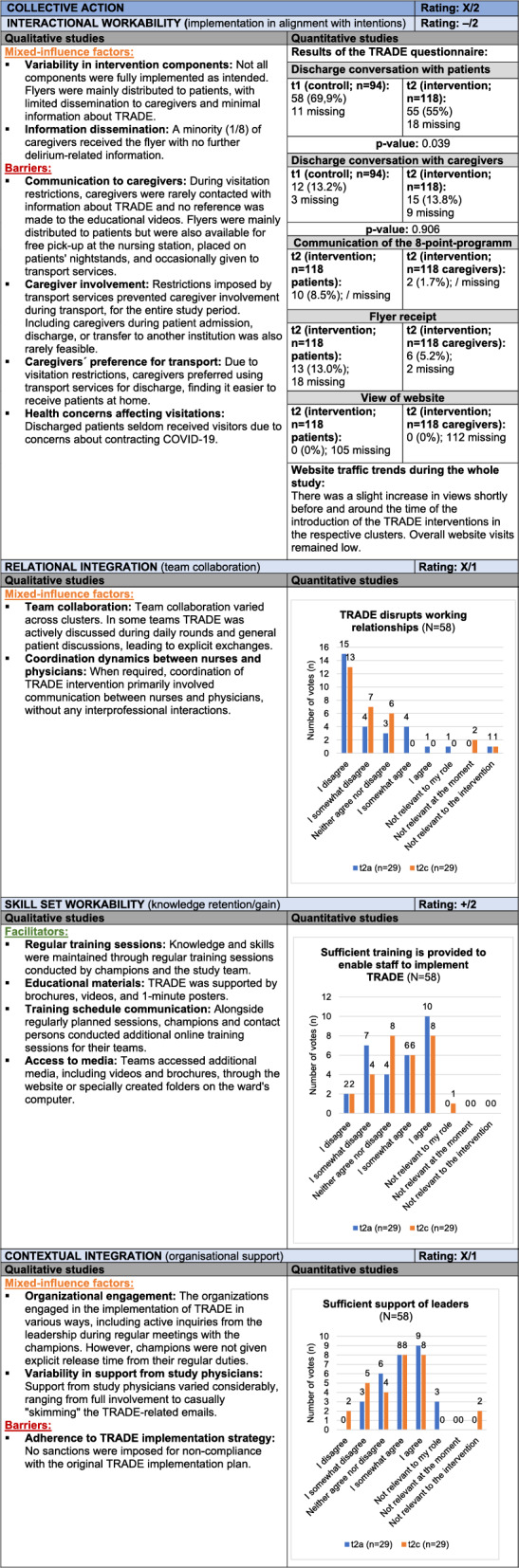
Joint display of *Collective Action*

Influence rating: + = positive influence (facilitator), – = negative influence (barrier), X = mixed influences, 0 = neutral; Strength of influence: 1 = weak influence, 2 = strong influence on implementation

#### Reflexive Monitoring

*Reflexive Monitoring* evaluates how participants assess the intervention to support continuous improvement, with subcategories *Systematization*, *Communal Appraisal*, *Individual Appraisal*, and *Reconfiguration*. Participants valued the program’s structure, with champions acting as reminders, though some found the email communication excessive. Practical application was challenged by pandemic-related resource constraints, and sharing delirium information with caregivers was hampered by time limits and communication issues. Suggestions for improvement included holding regular cluster meetings, expanding champion roles, and removing video passwords. Overall, this evaluation underscores the intervention's value and effective training materials, but identifies challenges concerning role clarity and limited acceptance, resulting in a mixed rating (X/2). Table 7 presents the joint display of *Reflexive Monitoring*, with example quotes from the qualitative data provided in Supplement 9.

**Table 7:**
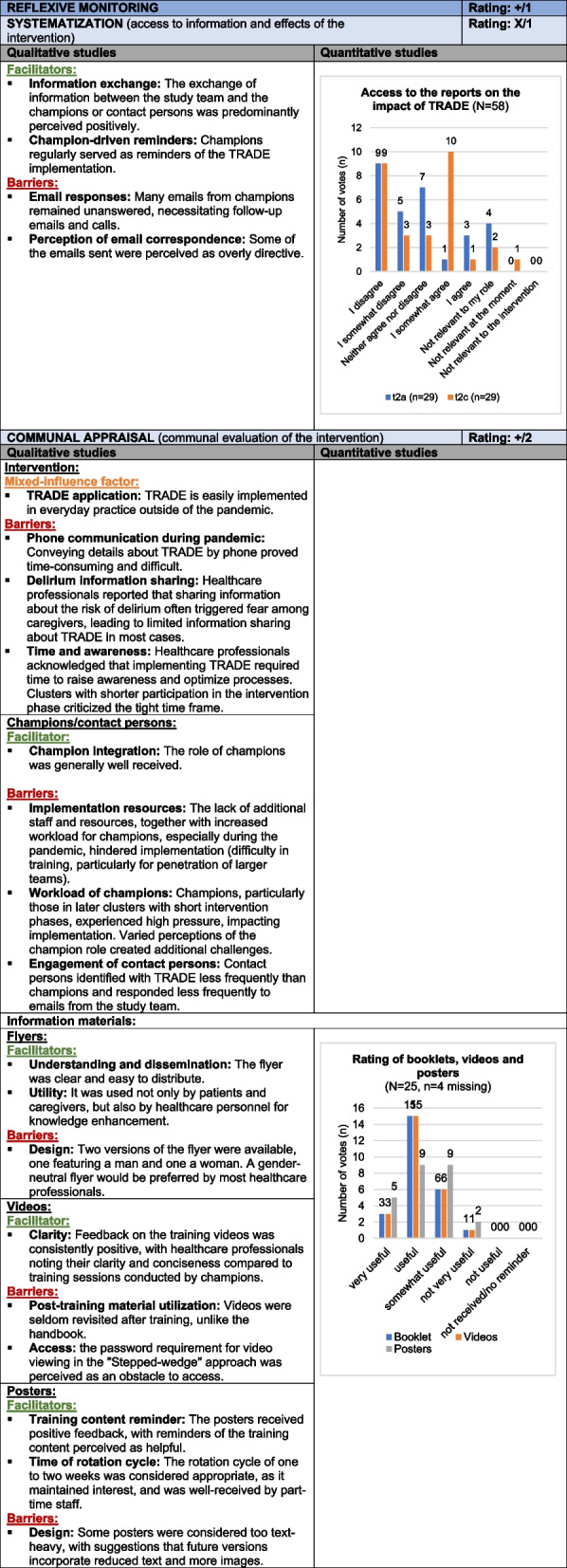

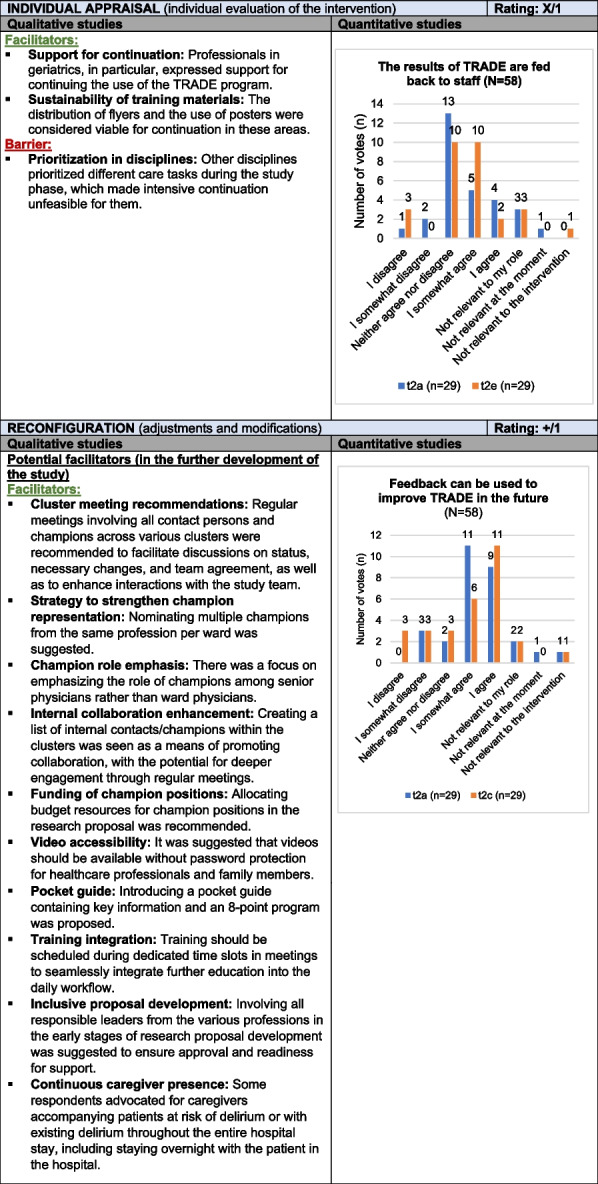
Joint display of *Reflexive Monitoring*

Influence rating: + = positive influence (facilitator), – = negative influence (barrier), X = mixed influences, 0 = neutral; Strength of influence: 1 = weak influence, 2 = strong influence on implementation

In summary, the *Implementation Mechanisms* highlight the intervention’s potential to foster shared understanding and practical knowledge, particularly in geriatrics. However, challenges in interprofessional collaboration, role clarity, and acceptance in other disciplines were identified. Pandemic restrictions and communication gaps further impacted engagement. Overall, the mixed influences (X/2) reflect strengths in training and materials, alongside challenges with structural and contextual barriers.

### Evaluation of Implementation Outcomes

*Implementation Outcomes* refer to the effects of the intervention across four categories: *Intervention Performance*, *Relational Restructuring*, *Normative Restructuring*, and *Sustainment (Normalization)*. The TRADE intervention led to heightened awareness of delirium prevention and prompted procedural changes, including collecting caregiver contact information and providing orientation aids during patient transfers. In one cluster, delirium management was restructured through a standardized anamnesis form and geriatric consultations, resulting on gradual, but not fully routinized, acceptance. Willingness to continue the intervention after the study was particularly high in geriatrics, with some professionals expressing interest in ongoing use of TRADE materials, especially for training new staff. Delirium prevention measures and active caregiver involvement were inconsistently applied across clusters. According to healthcare professional reports, this was partly due to limited engagement and challenges in integrating the intervention into daily routines. The term “study” also triggered skepticism in some cases. Additional contextual barriers included the impact of COVID-19 restrictions which limited caregiver involvement and interdisciplinary exchange. These aspects affected the depth of implementation.

Overall, procedural adjustments and training materials contributed positively to implementation, though inconsistencies in delirium prevention and contextual limitations hindered full adoption. Since the primary goal of the study was to test, rather than permanently establish, the intervention, the *Implementation Outcomes* receive a weak mixed rating (X/1). Table 8 presents the joint display of *Implementation Outcomes*, with example quotes from the qualitative data provided in Supplement 9.

**Table 8:**
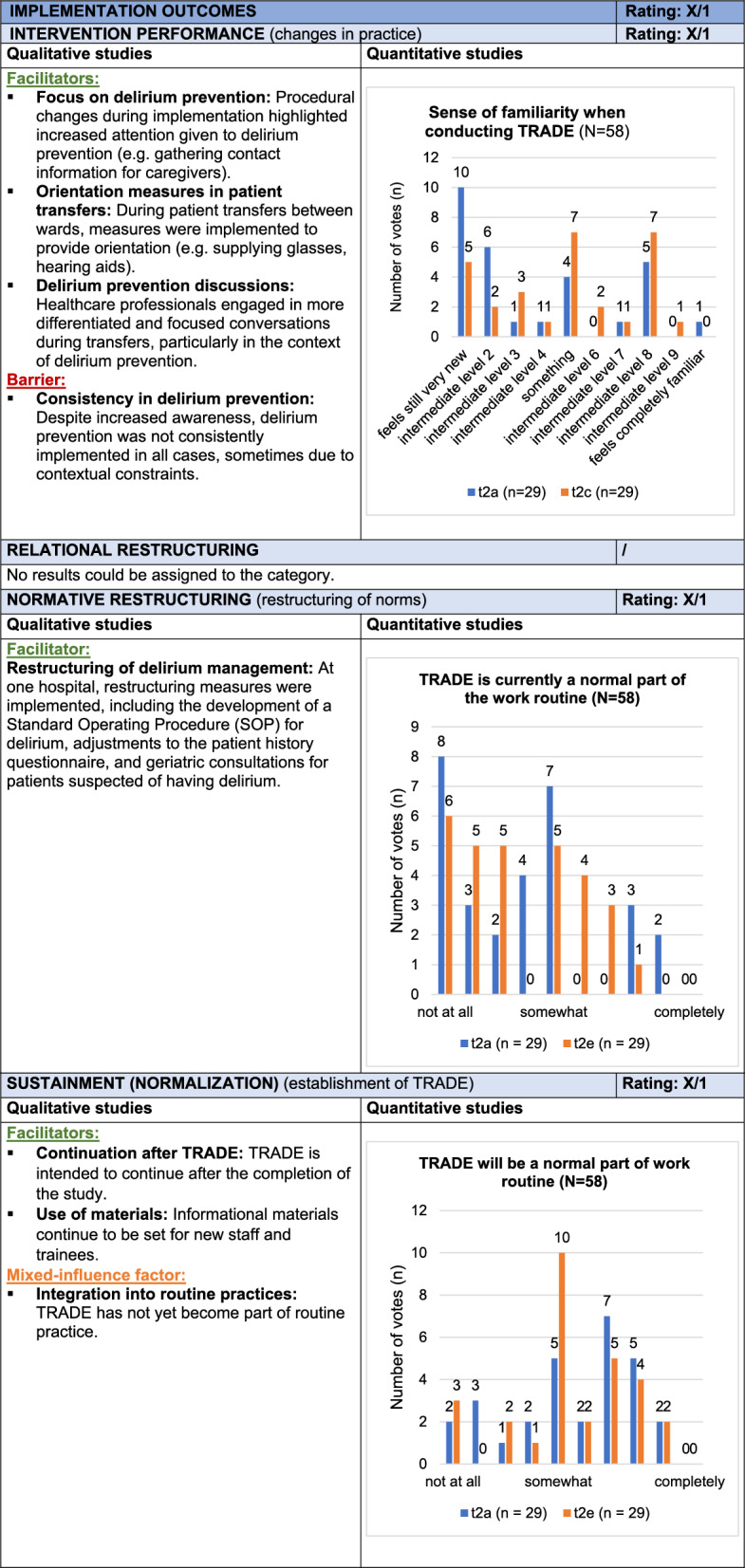
Joint display of *Implementation* O*utcomes*

Influence rating: + = positive influence (facilitator), – = negative influence (barrier), X = mixed influences, 0 = neutral; Strength of influence: 1 = weak influence, 2 = strong influence on implementation

### Evaluation of the TRADE *Logic Model*

**Fig. 1:**
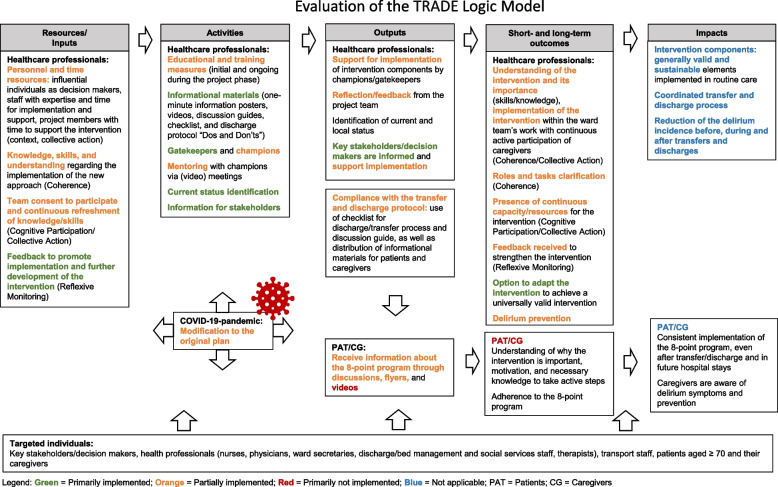
Evaluation of the logic model here

Some components of the Logic Model were effectively implemented, while others faced limitations due to varying levels of execution, from partial implementation to complete non-implementation (see Fig. 1). The "COVID-19 pandemic" was added as a factor to the model, given its substantial negative impact on the study, which restricted the full implementation of multiple components. Additionally,"role and task understanding" was included under "short- and long-term outcomes" as it proved essential to successful implementation. This factor warrants further attention in future iterations of the model. "Delirium prevention" was also relocated from "short- and long-term outcomes" to "impacts", and reframed as "reduction of delirium incidence during transfers and discharges". It is important to note that the primary objective of the trial, along with its accompanying process evaluation, was to explore the effects of the intervention and to assess the feasibility of the intervention rather than to achieve a long-term impact (4–10 years). Consequently, anticipated impacts such as routine integration of intervention components and consistently coordinated transfer and discharge processes have not yet been realized.

## Discussion

This study explored the implementation of a complex intervention designed to prevent delirium before, during and after discharge and transfer in hospitalized patients aged 70 years and older, using a mixed-methods approach aligned with current recommendations [29, 30]. This evaluation of the TRADE study examined the pre-developed program theory and its theoretical assumptions, identified contextual factors, facilitators, and barriers, and offered insights for clinical practice to improve care for older patients at risk of delirium, particularly during and after transfers.

The COVID-19 pandemic significantly influenced the implementation despite our adjustments, resulting in only partial realization of the intervention and implementation strategies within the clusters. The pandemic’s restrictions especially hindered our core strategy of involving caregivers in discharge and transfer processes by limiting their ability to accompany patients. Despite these challenges, healthcare professionals increased their focus on delirium prevention measures, including information gathering and orientation during transfers. Overall, the TRADE intervention received positive feedback from healthcare professionals.

The adverse effects of the pandemic on study operations, such as recruitment delays and scheduling disruptions, align with findings summarized in a review by Sathian et al. (2020) [50]. In addition to delays in elective procedures [51], our study showed that the pandemic also adversely affected standard care by limiting opportunities for communication with patients and caregivers. Pre-existing challenges, such as staff shortages, time constraints, and high turnover, known to challenge the implementation of new interventions before the pandemic [52], were further exacerbated. Visiting restrictions, while essential for infection control, also led to decreased orientation and emotional support for delirium-prone patients [53]. Similar studies have noted an increase in caregiver communication during visiting bans [54]. However, in our study, this contact was primarily phone-based and focused on issues arising rather than delirium risk. Caregivers found it difficult to assess the patient’s health over the phone, which made it challenging to communicate potential delirium risk. Additionally, although TRADE allowed for caregiver involvement in patient transport, transport services were preferred due to the pandemic’s logistical constraints. Unfortunately, caregivers were not permitted to accompany patients transported via these services during the pandemic.

Training sessions were shortened due to the COVID-19 situation. Instead of champions, we relied partially on contact persons and conducted some training ourselves. Despite these changes, the educational methods employed (e.g., training sessions, manuals, videos, and microlearning methods like 1-min posters [55]) were well-received. These methods accommodate different learning styles and allow for quick knowledge uptake [56], ensuring broad reach despite COVID-19’s constraints. While website traffic for information videos about the TRADE intervention was low, their availability on ward computers may have mitigated this issue for healthcare professionals.

Initially, ambiguity in roles and tasks posed challenges. Due to the pandemic, champions already burdened with substantial responsibilities struggled to take on additional study-related tasks due to workload and resource constraints. However, role clarity improved over time, aided by an extended implementation period, mentoring, and regular training. Although training was provided to all individuals, some clusters lacked champions, and in one case, nurses were not allowed to perform interventions. These findings indicate the need for stronger interprofessional collaboration and leadership support across interprofessional teams [57].

Beyond structural and logistical barriers, the process evaluation revealed perceptual and cultural challenges in delirium prevention. Some healthcare professionals expressed negative views about the term “study”, which may have contributed to limited participation. Previous research has also shown that delirium is often perceived by healthcare professionals as a low-priority issue or an unavoidable part of aging [58]. This study confirms that despite existing evidence and guidelines, delirium prevention remains inconsistently embedded in hospital care. While the intervention raised awareness and initiated change, especially in geriatrics, gaps persisted in other disciplines, such as cardiology. Given the high incidence of delirium in cardiology after major surgeries [59], raising delirium awareness across all disciplines is essential. These discrepancies reflect structural and cultural differences in how delirium is prioritized and highlight the importance of professional attitudes and perceived relevance for sustainable implementation.

The process evaluation also identified key shortcomings in delirium prevention management during discharge and care transitions. Patients and caregivers frequently reported receiving only preliminary discharge letters that lacked essential information about previous or current delirium and associated risks, information that is critical for continuity of care [60, 61]. Additionally, there was no shared understanding among healthcare professionals about who was responsible for communicating delirium-related information. Nurses often viewed this as a physician’s task, despite the fact that interprofessional collaboration is essential for effective delirium prevention [62].

Caregiver involvement emerged as a critical, yet underutilized component of delirium prevention. Although the intervention included informational materials and counseling tools that support understanding and management of delirium [63, 64], actual engagement was limited, partly due to pandemic-related restrictions and partly due to the absence of standardized integration procedures. Preparing caregivers in recognizing and responding to delirium risk [19, 65] should therefore be considered a routine component of discharge and transfer processes.

Structured, evidence-based care pathways for delirium prevention have been shown to reduce hospital length of stay and the risk of readmissions [66]. The TRADE intervention incorporated such structured elements to support coordination and continuity across care transitions. However, successful implementation requires early interprofessional planning and clearly defined responsibilities across clinical disciplines. These findings highlight the need to institutionalize delirium prevention as a shared, cross-professional responsibility that extends beyond hospitalization into transitional and post-acute care settings.

### Strengths and limitations

A key strength of this study is its theoretical and methodological contributions to complex intervention research, particularly through the application of a comprehensive mixed-methods process evaluation. Despite challenges such as the COVID-19 pandemic and limited time and resources, this approach provided a robust understanding of how and why the intervention functioned across different settings. The mixed-methods design not only allowed for in-depth exploration of implementation processes but also explained the limited adoption and adaptation of the intervention, addressing key barriers and facilitating recommendations for its further development [28, 29].

A distinguishing feature of this study was the use of joint displays to effectively integrate qualitative and quantitative data. These displays facilitated triangulation, enhanced validity, and enabled a nuanced understanding of the implementation mechanisms [67]. By visually presenting comprehensive datasets, the joint displays improved transparency and validity assessment, aligning with current recommendations for mixed-methods studies and complex intervention evaluations [30, 67]. Furthermore, these displays serve as a valuable methodological resource for advancing the integration and communication of mixed-methods research. Its application underscores the potential for broader adoption in nursing implementation research, in line with the latest UK MRC Framework for complex interventions [29], and highlights the value of rigorous mixed-methods designs for future nursing interventions.

Using the NPT as a theoretical framework further strengthened the study by guiding the conceptualization and evaluation of the implementation process. The NPT Coding Manual [42] supported our use of both parts A and B with relevant constructs. However, due to overlapping definitions, certain constructs were adapted to fit TRADE*-*specific contexts, which limited data assignment to some categories, such as *Relational Restructuring*.

An additional strength was the timing of our process data analysis which was conducted prior to analysing main study outcomes, in line with UK MRC framework recommendations [29].

The qualitative and quantitative data were collected across a wide range of professional roles, wards, and disciplines in four hospitals. While efforts were made to include all relevant interest holders and all healthcare professionals, the response rate to the NoMAD questionnaire was low, especially among physicians. This may affect external validity and limits the representativeness of the quantitative findings, particularly regarding interprofessional teamwork. Sensitivity analysis was not feasible due to the low number of participants at the second data collection point (t2e) and the variable intervention durations. Nonetheless, the comprehensive qualitative data provided detailed insights into implementation processes and participant experiences across clusters and disciplines, offering depth and context that strengthened the interpretation of the findings. The predominant alignment of qualitative and quantitative results further supports the overall validity of our conclusions. However, since the data reflect the implementation reality under pandemic conditions, generalizability should be interpreted with caution.

Including a diverse range of wards and disciplines provided further insights into the need for delirium prevention across clinical settings. However, the stepped-wedge design posed challenges. Staggering intervention timing across clusters required extensive training sessions, involvement of champions, contact persons and gatekeepers, as well as peer mentoring. Numerous interviews were conducted to achieve data saturation. Additionally, to prevent contamination in clusters not yet in the intervention phase, access to study videos on the study website was password-protected, which later proved to be a barrier.

Pandemic-related constraints affected various intervention components, including planned caregiver involvement through transportation services [25] which was limited due to pandemic regulations. Testing TRADE exclusively under pandemic conditions may influence the generalizability of our findings, as it remains unclear whether post-pandemic implementation willingness will match pre-pandemic levels. Additionally, the early stage of nursing intervention research in Germany may have presented further implementation challenges independent of the pandemic [68].

### Recommendation for further research and clinical practice

This study offers several recommendations to improve the implementation and effectiveness of interventions aimed at delirium prevention before, during and after discharges and/or transfers in older patients:

(i) Designating multiple champions per professional group and ward may enhance engagement and support the intervention’s sustainability by allowing task-sharing and mutual support. Allocating dedicated time for these roles is essential, as high workloads may limit champions' commitment [52].
(ii) Early involvement of all interest holders, including leaders across nursing, medical, and social work disciplines, should be prioritized to ensure commitment and interprofessional support. Engaging interest holders during the study design phase, ideally at the time of the funding application, can strengthen buy-in and help overcome implementation challenges [69].
(iii) Delirium awareness varies across disciplines. Therefore, it is essential to emphasize awareness, particularly in high-risk settings such as cardiology after major surgery [59]. Regular competency assessments, such as case vignettes, could help tailor training content to individual skill levels [70], allowing for targeted improvements in delirium prevention strategies.
(iv) Future interventions should facilitate caregiver involvement in discharge and transitions, even under restrictive conditions. Policies enabling caregiver participation, either in person or via alternative communication channels, can enhance patient orientation and emotional support, both critical for delirium prevention.
(v) Further research is needed to examine the intervention’s applicability outside pandemic contexts.

## Conclusion

Our process evaluation offers valuable insights for healthcare professionals seeking to improve discharge quality and transitions for older patients and their caregivers from acute care to home or post-acute institutions. Key implementation strategies, including the appointment of champions, targeted training sessions, and accessible informational resources, emerged as potentially effective for introducing new interventions. Strengthening interprofessional support and allocating dedicated time for champions could further enhance implementation, particularly in delirium prevention and caregiver involvement. Our comprehensive mixed-methods evaluation enhances understanding effective intervention implementation in complex healthcare settings. Addressing identified barriers and facilitators can improve delirium prevention and caregiver involvement, ultimately supporting smoother transitions and better outcomes for older patients during these critical phases of care.

## Supplementary Information

Supplementary Material 1. Supplementary file 1. Good Reporting of a Mixed Methods Study (GRAMMS).

Supplementary Material 2: Supplementary file 2. Interview guides (healthcare professionals, patients, caregivers, study physicians, study nurses).
Supplementary file 3. Status analysis.
Supplementary file 4: TRADE questionnaire (questions for the process evaluation).
Supplementary file 5. NoMAD questionnaire.
Supplementary file 6: Flowchart of the recruitment and exclusion process (TRADE questionnaire).
Supplementary file 7: Overview of the qualitative and quantitative methods.
Supplementary file 8. Visiting restrictions in clusters 1 to 4.
Supplementary file 9. Quotes from the interviews and focus groups.

Supplementary Material 3.

## Data Availability

The data supporting the findings of this study are not publicly available due to privacy concerns and the sensitive nature of the interview data. However, they can be obtained from the corresponding author upon reasonable request.

## Abbreviations

APN: Advanced Practice Nurse
CFIR: Consolidated Framework for Implementation Research
GRAMMS: Good Reporting of a Mixed Methods Study
HELP: Hospital Elder Life Program
LAR: Legally Authorized Representative
MRC: Medical Research Council
NoMAD: Normalization MeAsure Development
Nu-DESC: Nursing Delirium Screening Scale
NPT: Normalization Process Theory
TRADE: TRAnsport and DElirium of older people
3D-CAM: 3-Minute Diagnostic Interview for Confusion Assessment Method-defined Delirium
