# Supplementary material for: Implementation context, mechanisms and outcomes of a transitional care intervention to prevent delirium: a mixed-methods process evaluation from the TRADE study
Source: BMC Geriatr. 2025 Sep 25;25:704. doi: 10.1186/s12877-025-06331-8 (PMC12462268; doi:10.1186/s12877-025-06331-8)
Supplement: Supplementary file 1 — Supplementary Material 1. Supplementary file 1. Good Reporting of a Mixed Methods Study (GRAMMS). [file 12877_2025_6331_MOESM1_ESM.docx]

**Good Reporting of A Mixed Methods Study (GRAMMS) checklist**

| **No** | **Description** | **Where located in manuscript (page or appendix number)** |
| --- | --- | --- |
| 1 | Describe the justification for using a mixed methods approach to the research question. | Page 5 (Background) |
| 2 | Describe the design in terms of the purpose, priority and sequence of methods. | Page 9 (Design), pages 11-15 (Data Collection) |
| 3 | Describe each method in terms of sampling, data collection and analysis. | Pages 7-15 (Data Collection and Analysis) |
| 4 | Describe where integration has occurred, how it has occurred and who has participated in it. | Pages 15-16 (Synthesis) |
| 5 | Describe any limitation of one method associated with the present of the other method. | Pages 39-41 (Limitations) |
| 6 | Describe any insights gained from mixing or integrating methods. | Pages 16-35 (Results), pages 35-39 (Discussion) |

O'Cathain, A., Murphy, E., Nicholl, J. (2008). The quality of mixed methods studies in health services research. J Health Serv Res Policy, 13, 92-98. https://doi.org/10.1258/jhsrp.2007.007074
