# Supplementary material for: Implementation context, mechanisms and outcomes of a transitional care intervention to prevent delirium: a mixed-methods process evaluation from the TRADE study
Source: BMC Geriatr. 2025 Sep 25;25:704. doi: 10.1186/s12877-025-06331-8 (PMC12462268; doi:10.1186/s12877-025-06331-8)
Supplement: Supplementary file 2 — Supplementary Material 2: Supplementary file 2. Interview guides (healthcare professionals, patients, caregivers, study physicians, study nurses). Supplementary file 3. Status analysis. Supplementary file 4: TRADE questionnaire (questions for the process evaluation). Supplementary file 5. NoMAD questionnaire. Supplementary file 6: Flowchart of the recruitment and exclusion process (TRADE questionnaire). Supplementary file 7: Overview of the qualitative and quantitative methods. Supplementary file 8. Visiting restrictions in clusters 1 to 4. Supplementary file 9. Quotes from the interviews and focus groups. [file 12877_2025_6331_MOESM2_ESM.docx]

**Supplementary files (2-7, 9)**

[Supplementary file 2: Interview guides (healthcare professionals, patients, caregivers, study physicians, study nurses) 2](#_Toc189044205)

[Supplementary file 3: Status analysis 7](#_Toc189044206)

[Supplementary file 4: TRADE questionnaire (questions for the process evaluation) 16](#_Toc189044207)

[Supplementary file 5: NoMAD questionnaire 18](#_Toc189044208)

[Supplementary file 6: Flowchart of the recruitment and exclusion process (standardized questionnaire) 31](#_Toc189044209)

[Supplementary file 7: Overview of the qualitative and quantitative methods 32](#_Toc189044210)

[Supplementary file 9: Quotes from the interviews and focus groups 34](#_Toc189044211)

Supplementary file 2: Interview guides (healthcare professionals, patients, caregivers, study physicians, study nurses)

**Interview guide for healthcare professionals**

| **Key questions (with impulse questions):** |
| --- |
| **Training:**  If you think back to the time before the training...   - What were your thoughts about the study before you attended the training? - How well did you understand the study’s meaning and purpose beforehand? |
| **Training day and the time that followed:**  If you think back to the time during and after the training…   - How clearly was the intervention explained to you during the training?   - Prompt: How well did you understand the intervention’s purpose and meaning after the training?   - Prompt: How well did you understand your tasks or your role within the intervention after the training? - How useful did you find the training manuals?   - Prompt: How often did you use them after the training? - How useful did you find the training videos?   - Prompt: Did you revisit them after the training? - Do you have any specific comments or feedback about the training? |
| **Introduced intervention:**   - How well do you think the individuals involved in implementing the introduced intervention actually put it into practice in their daily work? - Putting yourself in your colleagues' shoes, what do you think they think of the intervention? |
| **Personal evaluation of the introduced intervention:**   - How successful was the training in supporting the practical implementation of the intervention?   - Prompt: To what extent did the training motivate employees to change/improve their work practices and put these changes into practice?   - Prompt: Did the training sessions adequately prepare employees for the intervention? If not, what additional training or changes would you have found beneficial? - What was your experience of the introduced intervention?   - Prompt: How closely did it match your expectations? - How feasible do you think it is to implement the intervention in practice?   - Prompt: To what extent are there times when it is difficult to implement it?   - Prompt: How practical has the introduced intervention been in practice?   - Prompt: To what extent was it possible to adhere to the planned intervention as taught in the training? If this was not possible, what modifications were made to overcome implementation issues or accommodate existing practices?   - Prompt: In your opinion, what factors negatively and positively influence feasibility? - What is your impression of the flyers and videos for the caregivers? (prompt: content, structure, appearance) - How did the COVID-19 pandemic impact TRADE? |
| **Review and sustainability of the intervention:**   - How much impact do you believe the introduced intervention has had? - How do you view the implementation of the intervention for the future?   - Prompt: How likely is it that the intervention can be continued as before?   - Prompt: Are there elements of the intervention that you believe can be easily maintained?   - Prompt: Are there elements of the intervention that are difficult to continue or are not functioning well? How could they be improved? - Online surveys were conducted, and the response rate was not optimal. Can you explain this? Would a paper-based survey potentially have had a higher response rate? |
| **Questions exclusively for champions/gatekeepers:**   - How much impact do you think the use of champions/gatekeepers has had?   - Prompt: How effective do you think you were in your role as a champion/gatekeeper?   - Prompt: How do you know how effective the roles have been?   - Prompt: Are there things you would have done differently in your role as champions/gatekeepers with hindsight?   - Prompt: Which elements do you remember as particularly effective?   - Prompt: How important do you consider your role as a champion/gatekeeper to be? - What experiences have you had with the tasks as a gatekeeper/champion? - What was your experience of peer mentor emails/phone calls?   - Prompt: How helpful were the regular peer mentor phone calls?   - Prompt: Did you wish anything different during the peer mentor phone calls, or would you have liked more support from the project team? If so, what? |
| **Questions exclusively for clinical staff (without gatekeeper/champion/leadership roles):**   - How helpful was the assigned champion to you? - How much influence did the leadership staff have on the intervention? |
| **Closing questions:**   - Are there any future developments or changes that you would wish for? - When you look back at all aspects of the intervention introduced as part of the TRADE study, what are your feelings about it? - Are there any elements or topics that have been neglected in your view? |

**Interview guide for patients (t1: during control phase (stepped-wedge); t2: during intervention phase):**

| **Hospital Stay:**  When you think back to your hospital stay, especially your discharge/transfer:   - To what extent were you, as a patient, involved in the discharge/transfer process?   - Prompt: What specific actions or conversations regarding your discharge/transfer took place during your time in hospital?   **Questions for patients during the intervention phase only (t1):**   - Did you or a family member/friend receive the flyer with the 8-point program? If yes: - What are your thoughts on the flyer and the 8-point program? - To what extent did you find the information and tips helpful? What would you have found helpful? - Did you watch the video on the topic of delirium? If yes: - What are your thoughts on the video? - To what extent did you find it helpful? What would you have found helpful l? |
| --- |
| **Direct Discharge/Transfer and Transportation:**   - How did the discharge and transportation process unfold in your case?   - Prompt: Did your caregiver/family member/friend come to the hospital right before your discharge?     - To what extent did you find this useful?   - Prompt: Were you accompanied by a family member or caregiver during transportation? - How useful did you find the presence of your family member or caregiver during transportation? |
| **New Facility/at Home:**   - How has your time been so far in the new facility or at home?   - Prompt: Specifically, how was the first day in the new facility or at home? - How did you feel about the first few days in the new facility or at home? What was particularly good or helpful for you? What didn't work well or wasn't helpful for you? - Was anybody with you during the first days in the new facility or at home?   - If yes, who was with you, and how did you feel about their presence? Did you find it useful?   - If no, would you have preferred someone to be there? - Prompt (only for individuals in a new facility): Are you able to have familiar items with you, such as photos, your own blanket, special clothing, books, etc.? - If yes, to what extent do you find these familiar items helpful? - If no, why not? - Prompt: Have you had visits from your loved ones or other people in the new facility or at home? - To what extent do you find these visits helpful and enjoyable? - How do you arrange these visits, and what do you like most about them? |
| **COVID-19:**   - In comparison to previous hospital stays, what impact did you experience in the hospital due to COVID-19? - Prompt: Could family members or loved ones visit you during your hospital stay? Were they involved in the discharge process or in accompanying you? - Prompt: What would you have desired in this situation? |
| **Closing questions:**   - Please tell me how you came to participate in the study (prompt: process, recruitment flyer): Why did you choose to participate in the study? Would you participate again, and if not, why not? - When you look back on the entire process from discharge until now, how do you feel about it? - Would you have liked more information, tips, or anything else for the period after your hospital stay? If so, what specifically? - Are there any important elements related to discharge and transfer that have not been addressed in your view? |

**Interview guide for caregivers (t1: during control phase (stepped-wedge); t2: during intervention phase):**

| **Hospital stay:**  When you think back to your mother’s/father’s hospital stay..., particularly their discharge:   - How involved were you, as a caregiver, involved in the discharge process?   - Prompt: What specific actions or conversations concerning the hospital discharge involved you as a family member?   - Prompt: Were you, as a caregiver, able to discuss the discharge?     - If yes, to what extent did you feel that the topics important to you, and potentially your loved one, were addressed and taken seriously?   **Questions for caregivers during the intervention phase only (t1):**   - Did you receive the flyer with the 8-point program? If yes:   - What are your thoughts on the flyer and the 8-point program?   - How helpful did you find the information and tips? What would you have found helpful? - Did you watch the delirium video on the website? If yes:   - What are your thoughts on the video?   - To what extent did you find it helpful? What would you have found helpful? |
| --- |
| **Direct discharge/transfer and transportation:**   - How did the discharge and transportation process unfold in your mother’s/father's/… case?   - Prompt: Were you or anybody else at the hospital immediately before the discharge of your mother/father/...?     - How important do you think it is to have someone with your mother/father/… before discharge? Did you find it helpful?   - Prompt: Did you or anybody else accompany your mother/father/… during transportation?     - If yes, how do you view another’s presence during transportation? To what extent did you find it useful? |
| **New facility/at home:**   - How has your mother’s/father's/… time been in the new facility or at home so far?   - Prompt: Specifically, how did the first day in the new facility or at home go?     - What was particularly good or helpful for you, and what was not successful or helpful?     - Were you or anybody else present during the admission to the new facility or at home, or on the admission day? If yes, who was present, and did you find it useful for your mother/father/...?   - Prompt (only for individuals in a new facility): Were you able to bring familiar items for your mother/father/..., such as photos, their own blanket, special clothing, books, etc.? If yes, to what extent do you find these familiar items helpful?   - Prompt: How often do you visit your mother/father/...? Do they receive other visits, and from whom?     - How helpful do you find these visits?     - How do you arrange and conduct these visits? |
| **COVID-19:**   - In comparison to previous hospital stays, what impact did COVID-19 have on your experience at the hospital?   - Prompt: Were you able to visit your family member in hospital during the COVID-19 pandemic? How involved were you in the discharge process?   - What would you have desired in this situation? |
| **Closing questions:**   - Please tell me how you came to participate in the study (process, recruitment flyer). - Why did you choose to participate in the study? Would you participate again, and if not, why not? - When you look back on the entire process from discharge until now, how do you feel about it? - Are there any important elements related to discharge and transfer that have not been addressed in your view? |

**Interview guide for study physicians**

| **Tasks:**   - What are your specific tasks as a study physician? |
| --- |
| **Training:**  Looking back at the training provided to clinical staff on the intervention measures...   - Did you participate in it?   If yes, continue here:  Looking back at the time during and after the training...   - - How did you find the explanations for the intervention during the training? How clearly was the intervention presented to you?     - Prompt: To what extent did you receive feedback on the training from your colleagues in the clinic?     - Prompt: What changes, if any, would you have desired?     - Prompt: Do you have any specific comments you would like to make about the training? |
| **Training of study nurses:**   - Did you participate in it?   If yes, continue here:   - - How did you find it? Did you have a clear understanding of what you and the study nurses should do afterwards?   - Looking back now, to what extent do you wish that anything had been different?   - Did you receive feedback on the training from the study nurses? If yes, what kind of feedback did you receive? |
| **Intervention:**   - What were your experiences with the introduced intervention?   - Prompt: To what extent do you think you and your colleagues actually implement the intervention in your daily practice?   - Prompt: What do you think are the reasons why the intervention could (not) be applied in practice as planned? Prompt: What, in your view, should have been done to better implement the intervention?   - Prompt: To what extent did you, in your role as a study physician, have to intervene? What measures did you take to enable the intervention?   - Prompt: What impact did the COVID-19 pandemic have on TRADE? - How do you view the effectiveness and implementation of the intervention for the future?   - Prompt: To what extent is it likely that the intervention can continue as before? |
| **Evaluation of Surveys:**   - How do you perceive the feasibility of recruitment/surveys with the patients and caregivers?   - Prompt: Is it possible to adhere to the surveys as presented in the training?   - Prompt: To what extent were changes introduced during the study phase to address implementation issues, etc.? - What factors do you believe negatively influence the feasibility of the surveys? What positively? - How do you assess the implementation of the surveys for further similar studies?   - Prompt: To what extent is it likely that these surveys can be conducted in the same way for further similar studies?   - Prompt: To what extent might individuals have difficulties conducting the surveys for some reason? - How do you assess the implementation of the intervention for further similar studies - TRADE 2 or similar studies? |
| **Support from the Project Team:**   - To what extent were you supported by the entire study team?   - Prompt: To what extent would you have wished for something different in terms of support? If yes, what would that be? |
| **Closing questions:**   - What do you think should have gone differently in TRADE? (If yes, what?) - When you look back on the entire TRADE study, how do you feel about it? - From your perspective, are there any aspects or topics that were not addressed but that you consider important? |

**Interview guide for study nurses:**

| **Training:**  When you think back to the training...   - To what extent did you find the explanations about the assessments and data collection forms during the training to be sufficient?   - Prompt: When the training was over, to what extent was it clear to you what you needed to do? - Do you have any specific comments you'd like to make about the training? |
| --- |
| **Feasibility of surveys (patients/caregivers):**   - This is now about recruiting patients and caregivers for the study. Please explain the recruitment process.   - Prompt: How do you identify potential patients/caregivers on the participating wards?   - Prompt: If you put yourself in the shoes of the other study nurses, what do you think they believe about the feasibility of the surveys? |
| **Self-assessment of the surveys:**   - To what extent did the surveys go as expected? - How do you perceive the feasibility of the surveys with patients and caregivers?   - Prompt: Is it possible to adhere to the surveys as presented in the training?   - Prompt: To what extent were changes introduced during the study phase to address implementation issues, etc.? - What factors, in your opinion, negatively influence the feasibility of the surveys? What positively? - Why did patients and caregivers agree to participate in the study, and why not? - To what extent did the recruitment flyer play a role in the recruitment? |
| **Conducting surveys for further studies:**   - How do you assess the implementation of the surveys for further similar studies?   - Prompt: To what extent is it likely that these surveys can be conducted in the same way for further similar studies?   - Prompt: To what extent might individuals have difficulties conducting the surveys for some reason?   - Prompt: Is there anything that, in your opinion, is not working well at the moment? If yes, how could it be improved? |
| **Contact persons/support:**   - To what extent were you supported by the entire study team?   - Prompt: To what extent did the on-site contact persons support you in the clinic?   - Prompt: To what extent would you have wished for something different in terms of support? If yes, what would that be? |
| **Closing questions:**   - When you look back on the entire TRADE study, how do you feel about it? - From your perspective, are there any aspects or topics that were not addressed but that you consider important? |

Supplementary file 3: Status analysis

**Status analysis in the TRADE Study**

Dear Gatekeeper of the TRADE Study,

As of (specific date), a collaboratively developed intervention to prevent delirium in the context of patient transfers and discharges will be introduced on your ward/in your hospital. An essential initial step in this process is a status analysis, which will assess the current status of discharge management and related processes and procedures on your ward/in your hospital. At the end of the project, this analysis will be conducted again to allow comparisons to be made.

In accordance with the German expert guidance "Discharge Management in Nursing"^[[1]](#footnote-1)^, we define discharge management as follows:

“Discharge management includes an early, systematic assessment of the individual needs of patients and their families; initiating and providing appropriate counseling, training, and coordination services; evaluating for appropriate post-stationary care; and facilitating internal transitions. The goal of discharge management is to offer patients and their families suitable and optimal preparation and support for their discharge or transfer, ensuring continuous care. Through coordination with post-discharge providers and information exchange between those involved in the discharge process, we aim to prevent gaps in care by effectively preparing patients and their families.”

We kindly request that you complete the enclosed questionnaire and return it to us. If you have any questions, please do not hesitate to contact the person responsible for this state analysis (Name/Contact Information of the person).

We sincerely thank you for your support!

Best regards,

The entire TRADE Team

| **Name of the hospital:** | |
| --- | --- |
| **Ward: ____________________________________**  **Fields of expertise:**  **Number of patient beds:**  **Number of staff:**  **Nurses:**  **Physicians:**  **Administrative staff:**  **Other:** | **Ward: ____________________________________**  **Fields of expertise:**  **Number of patient beds:**  **Number of staff:**  **Nurses:**  **Physicians:**  **Administrative staff:**  **Other:** |
| **Ward: ____________________________________**  **Fields of expertise:**  **Number of patient beds:**  **Number of staff:**  **Nurses:**  **Physicians:**  **Administrative staff:**  **Other:** | **Ward: ____________________________________**  **Fields of expertise:**  **Number of patient beds:**  **Number of staff:**  **Nurses:**  **Physicians:**  **Administrative staff:**  **Other:** |
| **Ward: ____________________________________**  **Fields of expertise:**  **Number of patient beds:**  **Number of staff:**  **Nurses:**  **Physicians:**  **Administrative staff:**  **Other:** | **Ward: ____________________________________**  **Fields of expertise:**  **Number of patient beds:**  **Number of staff:**  **Nurses:**  **Physicians:**  **Administrative staff:**  **Other:** |
| **Date:** | **Total time required to complete this state analysis:** |
| **Individuals involved in completing this state analysis, and their professional qualifications:** | |
| **Contact information of the person responsible for this state analysis (Gatekeeper):**  **Name:**  **Telephone number.: Email-address:** | |

| **Nr.** | **Questions** | **Responses** | | **Comments** |
| --- | --- | --- | --- | --- |
| **Procedures and regulations for discharge management** | | | | |
| 1.0 | Does the hospital have written, multiprofessional procedural regulation(s) for discharge management?  (Description of the necessary processes and professional framework conditions)^[[2]](#footnote-2)^ | **Please mark the appropriate option in the blank field:** | |  |
|  |  | **Yes** | **No** | **Comments:** |
|  |  |  |  |  |
| 1.1 | If yes, which procedural regulation(s) are in place (name all)?  Please send us the procedural regulation(s) together with the completed form (file via email or printed copy by post). | **Existing procedural regulation(s):** | | |
|  |  |  | | |

| **Nr.** | **Questions** | **Responses** | | **Comments** |
| --- | --- | --- | --- | --- |
| **Persons responsible for the discharge process** | | | | |
| 2.0 | Which individuals in the hospital are responsible for discharge management?  (planning and control competencies for individual discharge planning, transition support, and management) | **Please mark the appropriate option in the blank field:** | | **Comments:** |
|  |  | **Nurses** |  |  |
|  |  | **Physicians** |  |  |
|  |  | **Discharge management** |  |  |
|  |  | **Social workers** |  |  |
|  |  | **Administrative staff** |  |  |
|  |  | **Others** |  |  |
| 3.0 | Which individuals in the hospital are authorized to coordinate the discharge process?  (scheduling the discharge date, required actions, and handover in collaboration with the patient, family, and internal and external professional groups and institutions) | **Please mark the appropriate option in the blank field:** | |  |
|  |  | **Nurses** |  | **Comments:** |
|  |  | **Physicians** |  |  |
|  |  | **Discharge management** |  |  |
|  |  | **Social workers** |  |  |
|  |  | **Administrative staff** |  |  |
|  |  | **Others** |  |  |
| 4.0 | Which specific tasks do the respective individuals conducte during the entire discharge process? | **Responsible individuals and their tasks:** | | |

| **Nr.** | **Questions** | **Responses** | | **Comments** |
| --- | --- | --- | --- | --- |
| **Measures in discharge management – after the admission of patients** | | | | |
| 5.0 | Are data on discharge management systematically recorded? | **Yes** | **No** | **Comments:** |
|  |  |  |  |  |
| 5.1 | If yes, where are these data recorded? | **In the patient record** |  | **Comments:** |
|  |  | **In the designated discharge management program** |  |  |
|  |  | **Other** |  |  |
| 5.2 | If yes, which data are recorded by whom? | **Persons and data:** | | |
| 6.0 | Is an initial criteria-based assessment of post-stationary care needs performed within 24 hours of taking over patient care?  (Answer "No" if there is no initial assessment or if it is not conducted within the first 24 hours. The takeover of care may begin upon admission to the hospital or when taking over care of the patient on a general ward after transfer from an intensive care unit, the emergency room, or the operating room/recovery room.) | **Yes** | **No** | **Comments:** |
|  |  |  |  |  |
| 6.1 | If yes, which individuals perform this assessment? | **Please mark the appropriate option in the blank field:** | | **Comments:** |
|  |  | **Nurses** |  |  |
|  |  | **Physicians** |  |  |
|  |  | **Discharge management** |  |  |
|  |  | **Social workers** |  |  |
|  |  | **Administrative staff** |  |  |
|  |  | **Others** |  |  |
| 7.0 | Is a detailed assessment carried out when post-stationary care needs are identified?  (A detailed assessment is present when a need for post-stationary care and support has been determined; respond with "Yes" if a detailed assessment was conducted directly.) | **Yes** | **No** | **Comments:** |
|  |  |  |  |  |
| 7.1 | If yes, who conducts the assessment? | **Please mark the appropriate option in the blank field:** | | **Comments:** |
|  |  | **Nurses** |  |  |
|  |  | **Physicians** |  |  |
|  |  | **Discharge management** |  |  |
|  |  | **Social workers** |  |  |
|  |  | **Administrative staff** |  |  |
|  |  | **Others** |  |  |
| 8.0 | Are individual discharge plans carried out, including specific actions required for preparing post-stationary care?  (Presence of a written discharge plan listing the necessary interdisciplinary measures for individual discharge preparation.) | **Yes** | **No** | **Comments:** |
|  |  |  |  |  |
| 9.0 | Are demand-oriented information, advice, and training on post-stationary care and support needs available?  (Information/advice/training with the goal of strengthening patients/family members in individual competencies for the time after discharge.) | **Yes** | **No** | **Comments:** |
|  |  |  |  |  |
| 9.1 | If yes, who provides this information, advice, and training? | **Please mark the appropriate option in the blank field:** | | **Comments:** |
|  |  | **Nurses** |  |  |
|  |  | **Physicians** |  |  |
|  |  | **Discharge management** |  |  |
|  |  | **Social workers** |  |  |
|  |  | **Administrative staff** |  |  |
|  |  | **Others** |  |  |
| 10.0 | Does the institution have target group-specific information materials for preparing for discharge? | **Yes** | **No** | **Comments:** |
|  |  |  |  |  |
| 10.1 | If yes, which materials/for which target groups?  **Please include information materials.** | **Identification of target groups and materials** | | **Comments:** |
|  |  |  | |  |
| 11.0 | Does the institution assess patient and caregiver knowledge and skills in managing post-stationary care and support requirements?  (Assessment of knowledge and skills, from, for example, counseling and training situations or observations.) | **Yes** | **No** | **Comments:** |
|  |  |  |  |  |
| 12.0 | Is the discharge date coordinated with the patient and their caregivers at an early stage?  (early coordination/discussion of the planned discharge date with the patient) | **Yes** | **No** | **Comments:** |
|  |  |  |  |  |
| 13.0 | Is the discharge date coordinated with all relevant professional groups within the hospital?  (opportunity to coordinate the discharge date with all relevant professional groups) | **Yes** | **No** | **Comments:** |
|  |  |  |  |  |
| 14.0 | Is the discharge coordinated with external institutions or caregivers?  (coordination of discharge date and information exchange regarding the current (changed) situation/needs of the patient.) | **No** |  | **Comments:** |
|  |  | **Yes, with caregivers** |  |  |
|  |  | **Yes, with subsequent facilities/institutions** |  |  |
|  |  | **Others** |  |  |
| 15.0 | Is the adequacy of the discharge plan checked no later than 24 hours before discharge?  (Checking whether all planned measures have been implemented and whether these were sufficient from the perspective of the patients, i.e., "Yes" is marked if there has been a corresponding discussion between the responsible person at the hospital and the patient/family.) | **Yes** | **No** | **Comments:** |
|  |  |  |  |  |
| 15.1 | If yes, who performs the review? | **Please mark the appropriate option in the blank field:** | | **Comments:** |
|  |  | **Nurses** |  |  |
|  |  | **Physicians** |  |  |
|  |  | **Discharge management** |  |  |
|  |  | **Social workers** |  |  |
|  |  | **Administrative staff** |  |  |
|  |  | **Others** |  |  |

| **Nr.** | **Questions** | **Responses** | | **Comments** |
| --- | --- | --- | --- | --- |
| **Trainings** | | | | |
| 16.0 | On which topics have you attended training and professional development in the past 24 months? | **Person** | **Training sessions** | **Comments:** |
|  |  | **Nurses** |  |  |
|  |  | **Physicians** |  |  |
|  |  | **Discharge management** |  |  |
|  |  | **Social workers** |  |  |
|  |  | **Administrative staff** |  |  |
|  |  | **Others** |  |  |
| 16.1 | Whatadditional training is required and in which topics? |  | | **Comments:** |
| 16.2 | Which training measures are staff offered relating to discharge and delirium in the hospital? |  | | **Comments:** |

| **Nr.** | **Questions** | **Responses** | | **Comments** |
| --- | --- | --- | --- | --- |
| **Delirium** | | | | |
| 17.0 | Are there experts/contacts/representatives in the hospital for delirium? | **Yes** | **No** | **Comments:** |
|  |  |  |  |  |
| 17.1 | If yes, which professional group do they belong to? |  | | **Comments:** |
| 17.2 | If yes, have they received specific training/professional development on delirium? |  | | **Comments:** |
| 17.3 | If yes, which specific tasks do they have? |  | | **Comments:** |
| 18.0 | Have specific measures concerning delirium been taken at the hospital (e.g., working groups for staff, information events for patients/families, etc.)? | **Yes** | **No** | **Comments:** |
|  |  |  |  |  |
| 18.1 | If yes, which measures? |  | | **Comments:** |
| 18.2 | What is the time frame for these measures? |  | | **Comments:** |

| **Nr.** | **Questions** | **Responses** | | **Comments** |
| --- | --- | --- | --- | --- |
| **Impact of COVID-19 on discharge management** | | | | |
| 19.0 | Is COVID-19 currently affecting discharge management in the hospital/on the wards? | **Yes** | **No** | **Comments:** |
|  |  |  |  |  |
| 19.1 | If yes, please describe the impact, changes, and challenges as specifically as possible. |  | | **Comments:** |

| **Nr.** | **Questions** | **Responses** | | **Comments** |
| --- | --- | --- | --- | --- |
| **Procedures and implementation** | | | | |
| 20.0 | If you indicated above that you have a written, multiprofessional procedure for discharge management:  Do you implement this procedure in practice? | **Yes** | **No** | **Comments:** |
|  |  |  |  |  |
| 20.1 | If not, why not? Where are there deviations? |  | |  |

Supplementary file 4: TRADE questionnaire (questions for the process evaluation)

1. Did you know where Mr./Ms. [Name] would be discharged to?
   - Yes
   - No
2. When were you informed about the discharge date?
   - Not at all
   - Very shortly beforehand (<30 minutes)
   - A few hours beforehand
   - 1 day beforehand
   - 2 days beforehand
   - 2 days beforehand
3. Did you have a conversation about the discharge and further care arrangements (e.g., nursing, household, shopping, etc.)?
   - Yes
   - No

If yes, when did the conversation take place?

- - Very shortly beforehand (<30 minutes)
  - A few hours beforehand
  - 1 day beforehand
  - 2 days beforehand
  - 2 days beforehand

If yes, with whom did the conversation take place?

- - Discharge manager
  - Social services
  - Nursing staff
  - Physician
  - Other professional group: _______

If yes, how long did the conversation last?

- - <5 minutes
  - 5-10 minutes
  - 10-15 minutes
  - 15-20 minutes
  - Other: _______

Which topics were discussed during the conversation?

- - Further procedures (e.g., care arrangements, follow-up with the general practitioner, etc.)
  - Clarification of open questions
  - Other: _______

Did you need additional information?

- - Yes
  - No

If yes, what kind? ________________________________

Follow-up (caregivers)

1. Were the following topics from the 8-point-program discussed with you during your hospital discharge conversation?

- Support during the transfer process:
  - Support directly before discharge
  - Support during transportation
  - Prolonged support at the destination
- Creating familiarity (e.g. bringing familiar items, frequent visits)
- Providing information to facility staff (e.g., about medications, habits, needs for support, or consumption of alcoholic beverages)
- Promoting orientation (e.g. use of glasses and/or hearing aids, setting up calendars and clocks)
- Adjusting communication (e.g. speaking slowly and clearly)
- Structuring daily life (e.g. activities such as crossword puzzles, games, or reading newspapers
- Encouraging movement (e.g. increased walks or bed exercises)
- Stimulating eating and drinking

2. Did you receive a flyer about delirium in the hospital?

- Yes
- No

If yes, how did you receive it?

- Handed to you personally by the following professional group: _____
- Made available for pickup
- Other: _____

When did you receive the flyer?

- On the day of discharge
- One day before discharge
- 2-3 days before discharge
- Other: _____

Were the flyer’s contents new to you?

- Yes
- No

Did you watch the video?

- Yes
- No

Supplementary file 5: NoMAD questionnaire

**Survey to the TRADE** **Study (t2a)**

**Survey instructions**

Dear participant,

Today, we are reaching out to request your support. As part of the research project "TRADE - TRAnsport and DElir in older people", you are implementing the TRADE intervention in practice.

In this project, it is essential for us to understand how you, as hospital staff, assess the measures we have implemented in the discharge process. This helps us gain a better understanding of how our interventions are being applied and integrated on the wards. We therefore invite you to participate in this survey.

The questionnaire consists of 35 questions. Please allow approximately 10 minutes for completion.

For each statement, take time to decide which answer corresponds best to your experience and mark the appropriate circle or field.

All data is collected anonymously, cannot be attributed to you, and will be treated with the utmost confidentiality.

The survey will be conducted multiple times.

Thank you very much for your participation!

**Part A: Creating a Personal Code While Maintaining Anonymity**

In this first section, we kindly request that you create a personal code. As we will conduct the measurement repeatedly, the code allows us to compare the results at different measurement points while ensuring anonymity.

1. **Please enter your personal code, using your date of birth in the format MMYY and the first letter from the following: your first name, mother's first name, father's first name. For example, if your name is Karin Müller, your date of birth is 23.02.1967, your mother's name is Katharina, and your father's name is Bernd, your code would be: 0267kkb: 0267kkb**

**Part B: Questions about you**

1. **How many years have you been working for [Name of the organization/department]?**

If your department has merged with another or changed its name, please state the total time you've worked in that department (before and after the merger/name change).

- Less than 1 year
- 1 to < 3 years
- 3 to < 5 years
- 5 to < 10 years
- 10 to < 15 years
- More than 15 years

1. **I work on the following ward:**

- Ward (…)
- Ward (…)
- Ward (…)
- Across multiple wards (e.g., therapist)
- On other wards, namely: _____________________________

1. **How would you describe your professional activity? (Multiple choices possible)**

- Champion in the TRADE study **and …**
- Contact person in the TRADE study **and** **…**
- Registered nurse
- Nurse specialist/Advanced practice nurse
- Nursing assistant
- Physician
- Physiotherapist
- Administrative staff
- Counseling/Organization services:
  - Discharge management
  - Social services/workers
  - Admission/Patient management
- Other: _______________________________________________________________________________________

1. **Do you hold a managerial position in your stated professional role?**

- Yes
- No

1. **What is your highest professional qualification?**

- No vocational training
- Vocational training
- Bachelor
- Master/Diploma/Magister/State Examination
- Doctorate/PhD
- Habilitation

**Part C: Training and information event questions**

Training sessions and information events were provided before the beginning of the intervention phase. For individuals who did not attend any training or information events, we also made a presentation available for self-viewing. Please evaluate these offers in the following questions.

1. **Did you participate in a training session or information event and/or view the presentation for self-viewing?**

- Participated in a training session (duration approximately 1.5 hours)
- Participated in an information event (duration approximately 0.5 hours)
- Viewed the presentation provided by the study team
  - Desktop version on the station
  - Printed version
  - Version sent via email
  - Other: _________________
- I neither attended a training session or information event nor viewed the presentation.

1. **How would you rate the training or information event you attended or the presentation for self-viewing overall (grading from 1 = excellent to 6 = insufficient)?**

- 1 (excellent)
- 2 (good)
- 3 (satisfactory)
- 4 (sufficient)
- 5 (poor)
- 6 (insufficient)
- I neither attended a training session or information event nor viewed the presentation.

1. **What did you find particularly helpful and useful in the training, information event, or presentation for self-viewing?**
2. **What did you find unhelpful or not useful in the training, information event, or presentation for self-viewing?**
3. **How do you assess the "Guidelines for healthcare professionals in hospitals on involving caregivers”?**

**Very useful Somewhat useful not useful Don’t remember/not received**

**
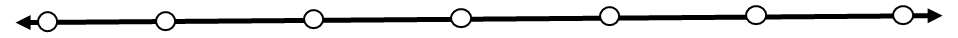
**

1. **Have you continued to use the "Guidelines for healthcare professionals in hospitals on involving caregivers" after the intervention implementation?**

- Yes
  - Yes, the entire guideline.
  - Yes, the basic knowledge about delirium.
  - Yes, the conversation guide.
  - Yes, the checklist for discharge or transfer.
- No
- Not received
- No longer remember the guidelines.

1. **How do you rate the videos that we showed you in the training/information session or provided in the presentation for your own review and on the TRADE website?**

**Very useful Somewhat useful not useful Don’t remember/not received**

**
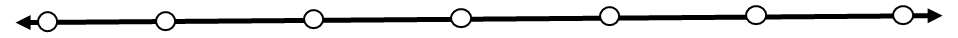
**

1. **Have you watched the videos again since the intervention was introduced?**

- Yes
- No
- No longer remember the videos.
- I have never seen the videos.

1. **How do you rate the 1-minute information posters (displayed on your ward)?**

**Very useful Somewhat useful not useful Don’t remember/no ward posters**

**
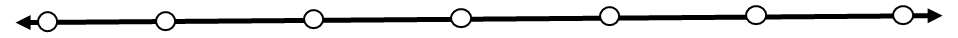
**

1. **Looking back, did you need more information? If yes, which information or informational materials would have been relevant for you?**

- No
- Yes, and specifically: ____________________________________________________________

**Part D: General questions about the TRADE intervention**

**Part E: Detailed questions about the TRADE interventions**

Part D and E are identical to the original NoMAD questionnaire and are therefore not included here.

**Survey to the TRADE Study (t2e)**

**Survey instructions**

Dear participant,

Today, we are reaching out to request your support.

As part of the research project "TRADE - TRAnsport and DElirium in older people", you are implementing the TRADE intervention in practice.

For the project, it is now important for us to understand how you, as clinic staff, assess the measures we have implemented in the discharge process. This helps us gain a better understanding of how our interventions are being applied and integrated on the wards. We invite you to participate in the survey.

The questionnaire consists of 33 questions. Please take about 10 minutes to complete it.

For each statement, take time to decide which answer corresponds best to your experience and mark the appropriate circle or field.

All data will be collected anonymously and cannot be attributed to you, and will be treated with the utmost confidentiality.

Thank you for your participation!

**Part A: Creating a personal code while maintaining anonymity**

In this first section, we kindly request that you create a personal code. As we will conduct the measurement repeatedly, the code allows us to compare the results at different measurement points while ensuring anonymity.

1. **Please enter your personal code, using your date of birth in the format MMYY and the first letter from the following: your first name, mother's first name, father's first name. For example, if your name is Karin Müller, your date of birth is 23.02.1967, your mother's name is Katharina, and your father's name is Bernd, your code would be: 0267kkb: 0267kkb**

**Part B: Questions about you**

1. **How many years have you been working for [Name of the organization/department]?**

If your department has merged with another or changed its name, please state the total time you've worked in that department (before and after the merger/name change).

- Less than 1 year
- 1 to < 3 years
- 3 to < 5 years
- 5 to < 10 years
- 10 to < 15 years
- More than 15 years

1. **I work on the following ward:**

- Ward (…)
- Ward (…)
- Ward (…)
- Across multiple wards (e.g., therapist)
- On other wards, namely: _____________________________

1. **How would you describe your professional activity? (Multiple choices possible)**

- Champion in the TRADE study **and …**
- Contact person in the TRADE study **and** **…**
- Registered nurse
- Nurse specialist/Advanced practice nurse
- Nursing assistant
- Physician
- Physiotherapist
- Administrative staff
- Counseling/Organization services:
  - Discharge management
  - Social services/workers
  - Admission/Patient management
- Other: _______________________________________________________________________________________

1. **Do you hold a managerial position in your stated professional role?**
   1. Yes
   2. No
2. **What is your highest professional qualification?**

- No vocational training
- Vocational training
- Bachelor
- Master's/Diploma/Magister/State Examination
- Doctorate/PhD
- Habilitation

**Part C: Questions about the information material**

1. **Do you still use the "Guidelines for healthcare professionals in the hospital for involving caregivers"?**

- Yes
  - Yes, the entire guideline.
  - Yes, the basic knowledge about delirium.
  - Yes, the conversation guide.
  - Yes, the checklist for discharge or transfer.
- No
- Did not receive.
- No longer remember the guideline.

1. **Have you watched the videos again after the intervention was introduced?**

- **Yes**
- **No**
- **Don’t remember the videos.**
- **I have never seen the videos.**

1. **How do you rate the 1-minute information posters (on your ward)?**

**Very useful Somewhat useful not useful Don’t remember/not seen**

**
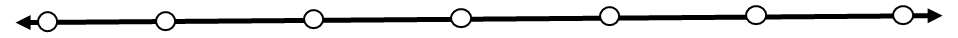
**

1. **With hindsight, did you need more information? If yes, which information or informational materials would have been relevant to you?**

- No
- Yes, specifically: _______________________________________________

**Part D: General questions about the TRADE** **intervention**

**Part E: Detailed questions about the TRADE interventions**

Part D and E are identical to the original NoMAD questionnaire and are therefore not included here.

Supplementary file 6: Flowchart of the recruitment and exclusion process (standardized questionnaire)

n = 396 Recruited participants

C: 181; I = 215

n = 80 Participants with early discontinuation

n = 104 Excluded participants with suspected delirium or missing delirium information

n = 212 Participants (Process evaluation)

C: 94; I = 118

Legend: C = Control group; I = Intervention group

Supplementary file 7: Overview of the qualitative and quantitative methods

| **Qualitative data** | | | |
| --- | --- | --- | --- |
| **Methods** | **Timepoints** | **Participants** | **Total time (min.–max.) for interviews/focus groups in minutes** |
| **Interviews with patients and caregivers (n = 33)** | t1 (n = 16) | n = 10 Patients  n = 6 Caregivers | 364 (9**–**49) |
|  | t2 (n = 17) | n = 9 Patients  n = 8 Caregivers | 600 (22**–**66) |
| **Interviews with healthcare professionals**  **(n = 39)** | t2c  (n = 39) | n = 5 Nurses  n = 2 Physicians  n = 2 Administrative staff  n = 1 Social workers  n = 9 Champions (4 nurses, 3 physicians, 2 social workers)  n = 5 Contact persons (2 nurses, 1 patient management,1 physiotherapist, 1 speech/occupational therapist)  n = 4 Gatekeeper (4 physicians)  n = 4 Nursing directors  n = 4 Study nurses  n = 3 Study physicians | 1522 (20**–**66) |
| **Focus groups (n = 2)** | t2c (n = 5) | n = 3 Contact persons (3 nurses)  n = 2 Medical directors | 81 (40**–**41) |
| **Documents**  **(n = 82)** | t0-t3 | n = 36 Protocols of meetings with clusters  n = 32 Protocols of meetings with project partners  n = 4 Protocols of meetings with champions and contact persons (each cluster/one protocol for entire study period)  n = 10 Other protocols (e.g. preliminary discussions with ward managers) |  |
| **Status analysis**  **(n = 14)** | t0 | n = 7 |  |
|  | t3 | n = 7 |  |
| **Quantitative data** | | | |
| **Methods** | **Timepoints** | **Participants** | |
| **TRADE questionnaire (n = 424)** | t1 (n = 188) | n = 94 Patients  n = 94 Caregivers | |
|  | t2 (n = 236) | n =118 Patients  n = 118 Caregivers | |
| **NoMAD**  **(n = 58)** | t2a (n = 29) | n = 18 Nurses (n=2 champions, and n=2 contact persons)  n = 1 Advanced practice nurse  n = 1 Nursing assistant  n = 1 Physician in training  n = 1 Specialist physician  n = 2 Senior physicians  n = 1 Administrative staff  n = 1 Physiotherapist  n = 1 Speech therapist and champion  n = 1 Social services  n = 1 Others: Nursing service manager  Note: NoMAD not available for occupational therapist, discharge and bed managers. | |
|  | t2b | / | |
|  | t2c | / | |
|  | t2e (n = 29) | n = 15 Nurses (n=1 champion, n=5 contact persons)  n = 1 Advanced practice nurse  n = 4 Physicians in training  n = 2 Senior physicians (n=1 contact person)  n = 2 Administrative staff  n = 1 Physiotherapist  n = 1 Speech therapist (n=1 champion)  n = 2 Social services  n = 1 Others: Nursing service manager  Note: NoMAD not available for nursing assistants, specialist physicians, occupational therapists, discharge and bed managers. | |

**Legend:** t0: Pre-study; t1: Control phase; t2: Intervention phase, with t2a: Intervention weeks 4 and 5; t2b: Intervention weeks 11 and 12; t2c: Intervention weeks 12 to 20; t2d: Intervention weeks 29 and 30; t2e: 2 weeks before the end of the study (Intervention weeks: Cluster 1 weeks 38 and 39, Cluster 2 weeks 32 and 33, Cluster 3 weeks 26 and 27, Cluster 4 weeks 20 and 21); t3: Post-study

Supplementary file 9: Quotes from the interviews and focus groups

| Implementation Context | |
| --- | --- |
| Reframing Organizational Logics | *"If someone came to us and said, 'Yes, we are conducting a study, and we need patients,' we always thought, 'Oh, help, help. What does that mean?' Because unfortunately, we have already had some not-so-great experiences in that regard."* (Contact person_11, Pos. 154) |
| Strategic Intentions | *„I'll mention the usual problems: staff shortage, high stress levels. Patients in and out quickly [of the hospital].“* (MA_32, Pos. 38) |
| Negotiating Capacity | *"On the wards, there are also posters on 'What is TRADE [project name; anonymized].' So, you can stop and read them. Unfortunately, due to the visitor ban, the effect has been completely eliminated now." (Contact person_11, Pos. 108)* |
| Adaptive Executions | *"We have a complete visiting ban starting today. (...) It also doesn't make it easier that you have to somehow communicate with caregivers via telephone."* (Contact person_11, Pos. 84) |
| Implementation Mechanism | |
| Coherence | |
| Differentiation | *"We did delirium prevention before. It’s just the issue wasn't emphasized."* (Champion_33, Pos. 24) |
| Communal Specification | Champions about training: *"(...) it was also very easy to understand. I also have to be completely honest; I mean, coming from the nursing profession, this is of course already a topic that we come across. (...) Therefore, there was nothing specific that I had to delve into again."* (Champion_33, Pos. 14)  Champion about team training*: "I took this training. I also engaged in discussions with people."* (Champion_33, Pos. 46) |
| Individual Specification | *"Exactly, that's what it's about – making caregivers aware and encouraging them to keep an eye on things, asking themselves, 'What does my family member need to avoid slipping into this phase?' "*(Champion_33, Pos. 76) |
| Internalization | *"(...) when appointed as a champion to coordinate everything, to meet regularly, and discuss, and so forth."* (Champion_33, Pos. 60) |
| Cognitive Participation | |
| Initiation | *"(...) because, as departmental management, we consistently inquire about the current status of TRADE [project name; anonymized].”* (Leader_21, Pos. 32) |
| Enrolement | *"And then, in the end, I agreed that we would send the employees to the training once, but beyond that, we cannot actively participate at this time."* (Leader_12, Pos. 8) |
| Legitimation | *"We should do it [TRADE [project name; anonymized]]. And we know, we are at a university hospital, and studies are a topic, and that's a good thing too."* (Champion_41, Pos. 126) |
| Activation | *"It's [TRADE [project name; anonymized]] not denied, it is supported."* (Contact person 13, Pos. 136) |
| Collective Action | |
| Interactional Workability | *"On our ward, the flyers were kept at the reception desk with our ward administrative person. She distributed them whenever a patient was admitted."* (Contact person_25, Position 54) |
| Relational Integration | *"We just don't have a proper interface between professional groups."* (Champion_33, Pos. 56) |
| Skill Set Workability | *"(…) So there’s an extra folder where everyone on the station team has access to various files (…). So, a folder labelled 'TRADE [project name; anonymized] Study'. And the video was included there, where you can always go back to it. In case you need something quickly."* (Champion_41, Pos. 92) |
| Contextual Integration | *“The head of nursing repeatedly pointed it out to me, and we also have our regular meetings where we discuss internal matters. It was occasionally a topic there, where she would follow up: 'How is it going?' Yes, but that was pretty much it."* (Champion_33, Pos. 162) |
| Reflexive Monitoring | |
| Systematization | *“I always noticed that when I addressed TRADE [project name; anonymized] with colleagues in our meeting, 'Oh yes, that's right. That exists.' So, it was another reminder. That was certainly helpful."* (Contact person_11, Pos. 144) |
| Communal Appraisal | Quote – Intervention: *"(...) that it is rather difficult to implement TRADE [project name; anonymized] at the moment."* (Contact person_24, Pos. 30)  Quote – Champions*: "Would it have been helpful if several champions had been deployed?” B: “Yes, I believe so."* (Champion_41, Pos. 217-220)  Quotes – Information material for teams: *"So, they are still available. But I think in the beginning, more employees looked at them, but now they look at them less because they are already familiar with them."* (Contact person_25, Pos. 92)  Quote – Information material for patients and caregivers: *"Well, I think, basically, it's certainly helpful to try it in different ways. (...) I’m sure, there are some who also benefit a lot from the video, and some, I think, prefer to read through it. But I think offering both is optimal."* (Employee_11, Pos. 46) |
| Individual Appraisal | *"I think that the dissemination of informational materials, the nursing aspect/I believe our task would be, for example, to clearly state as early as possible – which is not always possible – when the discharge date is, please start the interventions now. And to make sure that this is definitely standardized and coordinated through the administrative staff."* (Employee_11, Pos. 108) |
| Reconfiguration | *"I believe that if it could be more firmly integrated into the process, it could become more established as a routine. Then I believe, yes."* (MA_11, Pos. 101-102)  *"Did you have any more need for conversation?" "Yes, at least to have a point of contact when you feel the need to talk or inquire about something. The moment you are away from the hospital, the umbilical cord is cut. (...) But at the moment you are away, it's over."* (Caregiver_with_11, Pos. 225-226) |
| Implementation Outcome | |
| Intervention Performance | *"So, we now have buttons for our patient board. These are simply magnets with 'TRADE' [project name; anonymized] written on them, and we mark all potential patients over 70 years old."* (Champion_41, Pos. 102) |
| Relational Restructuring | / |
| Normative Restructuring | *“Some colleagues also wanted to have that for themselves, for the documents, because it's so nice for reference. (...) So, I used it for new staff. As an aid with geriatric patients or delirium patients, dementia patients."* (Champion_42, Pos. 16-18) |
| Sustainment | *"So, I think there has definitely been an awareness that has developed over time with this issue. We've also talked about it occasionally. So, I don't believe it's completely ineffective."* (Contact person_24, Pos. 26) |

1. Deutsches Netzwerk für Qualitätsentwicklung in der Pflege (2019) *Expertenstandard Entlassungsmanagement in der Pflege [Expert Standard for Discharge Management in Nursing]*, 2nd edn. Schriftenreihe des Deutschen Netzwerks für Qualitätsentwicklung in der Pflege, Osnabrück. [↑](#footnote-ref-1)
2. The text in light gray serves as an explanation. [↑](#footnote-ref-2)
