# Supplementary material for: Implementation context, mechanisms and outcomes of a transitional care intervention to prevent delirium: a mixed-methods process evaluation from the TRADE study
Source: BMC Geriatr. 2025 Sep 25;25:704. doi: 10.1186/s12877-025-06331-8 (PMC12462268; doi:10.1186/s12877-025-06331-8)
Supplement: Supplementary file 3 — Supplementary Material 3. [file 12877_2025_6331_MOESM3_ESM.pptx]

## Slide 1
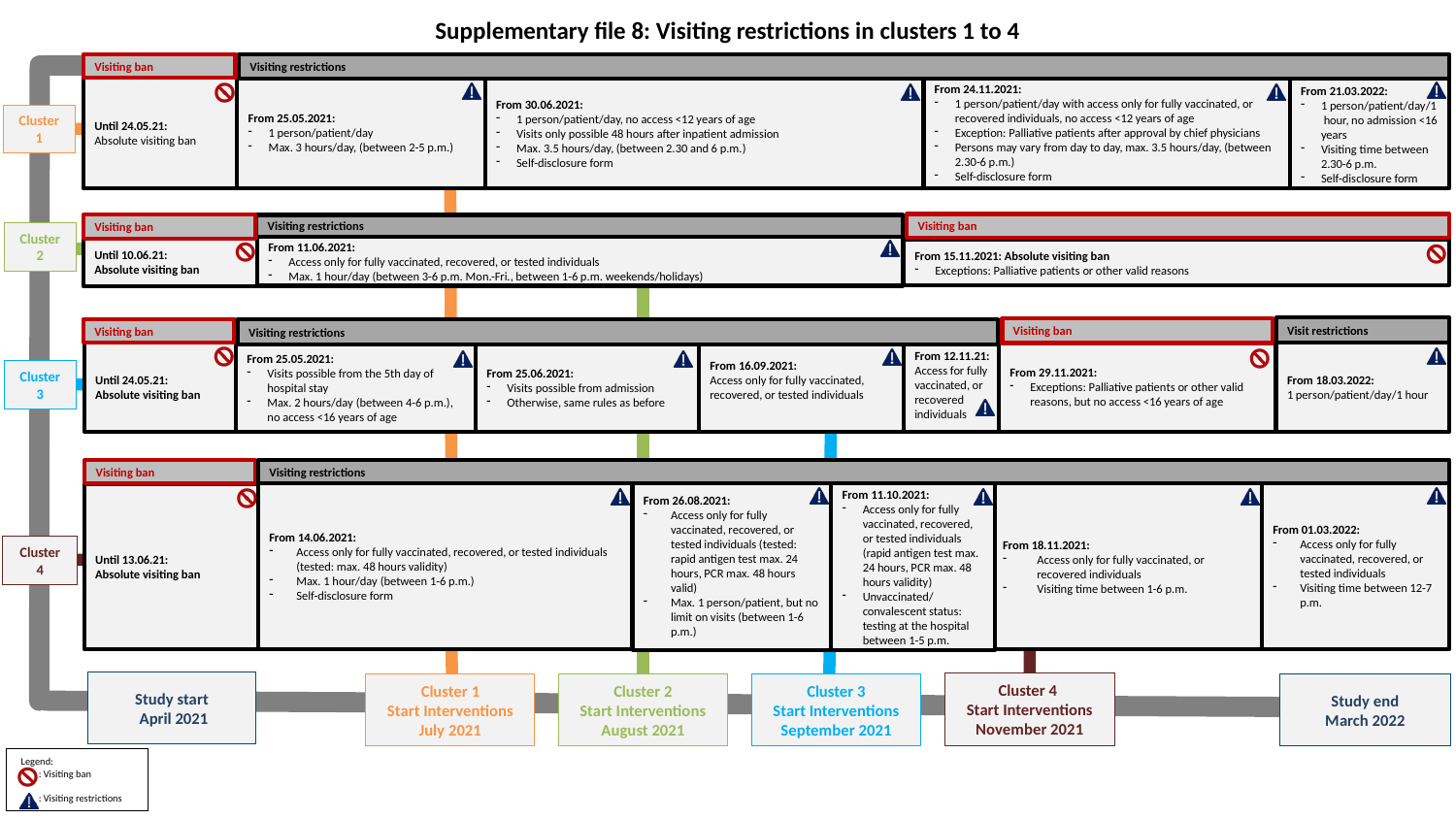

Supplementary file 8: Visiting restrictions in clusters 1 to 4
Visiting restrictions
Visiting ban
From 24.11.2021:
1 person/patient/day with access only for fully vaccinated, or recovered individuals, no access <12 years of age
Exception: Palliative patients after approval by chief physicians
Persons may vary from day to day, max. 3.5 hours/day, (between 2.30-6 p.m.)
Self-disclosure form
From 25.05.2021:
1 person/patient/day
Max. 3 hours/day, (between 2-5 p.m.)
Until 24.05.21:
Absolute visiting ban
From 21.03.2022:
1 person/patient/day/1 hour, no admission <16 years
Visiting time between 2.30-6 p.m.
Self-disclosure form
From 30.06.2021:
1 person/patient/day, no access <12 years of age
Visits only possible 48 hours after inpatient admission
Max. 3.5 hours/day, (between 2.30 and 6 p.m.)
Self-disclosure form
Cluster 1
Visiting ban
Visiting ban
Visiting restrictions
Cluster
2
From 11.06.2021:
Access only for fully vaccinated, recovered, or tested individuals
Max. 1 hour/day (between 3-6 p.m. Mon.-Fri., between 1-6 p.m. weekends/holidays)
Until 10.06.21:
Absolute visiting ban
From 15.11.2021: Absolute visiting ban
Exceptions: Palliative patients or other valid reasons
Visit restrictions
Visiting ban
Visiting ban
Visiting restrictions
From 29.11.2021:
Exceptions: Palliative patients or other valid reasons, but no access <16 years of age
From 18.03.2022:
1 person/patient/day/1 hour
Until 24.05.21:
Absolute visiting ban
From 16.09.2021:
Access only for fully vaccinated, recovered, or tested individuals
From 12.11.21:
Access for fully vaccinated, or recovered individuals
From 25.05.2021:
Visits possible from the 5th day of hospital stay
Max. 2 hours/day (between 4-6 p.m.), no access <16 years of age
From 25.06.2021:
Visits possible from admission
Otherwise, same rules as before
Cluster
3
Visiting restrictions
Visiting ban
From 26.08.2021:
Access only for fully vaccinated, recovered, or tested individuals (tested: rapid antigen test max. 24 hours, PCR max. 48 hours valid)
Max. 1 person/patient, but no limit on visits (between 1-6 p.m.)
From 01.03.2022:
Access only for fully vaccinated, recovered, or tested individuals
Visiting time between 12-7 p.m.
From 14.06.2021:
Access only for fully vaccinated, recovered, or tested individuals (tested: max. 48 hours validity)
Max. 1 hour/day (between 1-6 p.m.)
Self-disclosure form
From 11.10.2021:
Access only for fully vaccinated, recovered, or tested individuals (rapid antigen test max. 24 hours, PCR max. 48 hours validity)
Unvaccinated/ convalescent status: testing at the hospital between 1-5 p.m.
From 18.11.2021:
Access only for fully vaccinated, or recovered individuals
Visiting time between 1-6 p.m.
Until 13.06.21:
Absolute visiting ban
Cluster
4
Study start
 April 2021
Cluster 1
Start Interventions
July 2021
Study end
March 2022
Cluster 2
Start Interventions
August 2021
Cluster 3
Start Interventions
September 2021
Cluster 4
Start Interventions
November 2021
Legend:
 : Visiting ban
 : Visiting restrictions
